# Supplementary material for: MAPK-pathway inhibition mediates inflammatory reprogramming and sensitizes tumors to targeted activation of innate immunity sensor RIG-I
Source: Nat Commun. 2021 Sep 17;12:5505. doi: 10.1038/s41467-021-25728-8 (PMC8448826; doi:10.1038/s41467-021-25728-8)
Supplement: Supplementary file 1 — Supplementary Information [file 41467_2021_25728_MOESM1_ESM.pdf]

Supplementary Information

MAPK-mediated inflammatory reprogramming sensitizes tumors to targeted activation of innate immunity sensor RIG-I

Brägelmann et al.

Supplementary Methods

Antibodies used for immunoblotting

Primary antibodies used: Actin (#sc-47778, Santa Cruz Biotechnology, 1:5000), pan-AKT (#2920, Cell Signaling, 1:1000), $pAKT^{S473}$ (#9271, Cell Signaling, 1:1000), BCL2 (#2876, Cell Signaling, 1:1000), $BRAF^{V600E}$ (#E19290, Spring Bioscience, 1:1000), Cas9 (#14697, Cell Signaling, 1:1000), cleaved Casp.3 (#9664, Cell Signaling, 1:1000), cPARP (#55-2597, BD Bioscience, 1:1000), EGFR (#2239, Cell Signaling, 1:1000), $pEGFR^{Y1068}$ (#3777, Cell Signaling, 1:1000), ERK (#4696, Cell Signaling, 1:1000), $pERK^{T202/Y204}$ (#4370, Cell Signaling, 1:1000), H3K27me3 (#9733, Cell Signaling, 1:1000), H3K9me3 (#C15410056, Diagenode, 1:1000), Hsp90 (#4877, Cell Signaling, 1:5000), IFIT1 (#14769, Cell Signaling, 1:1000), IRF1 (#8478, Cell Signaling, 1:1000), IRF3 (#4302, Cell Signaling, 1:1000), $pIRF3^{S396}$ (#4947, Cell Signaling, 1:1000), MAVS (#3993, Cell Signaling, 1:1000), MCL1 (#5453, Cell Signaling, 1:1000), p16 (#ab108349, Abcam, 1:1000), p21 (#2947, Cell Signaling, 1:1000), p27 (#3686, Cell Signaling, 1:1000), p65 (#8242, Cell Signaling, 1:1000), $pp65^{S5367}$ (#3033, Cell Signaling, 1:1000), RIG-I (#3743, Cell Signaling, 1:1000), STING (#13647, Cell Signaling, 1:1000), TBK1 (#3504, Cell Signaling, 1:1000), $pTBK1^{S172}$(#5483, Cell Signaling, 1:1000), total H3 (#C15310135, Diagenode, 1:1000), VTCN1 (#14572, Cell Signaling, 1:1000).

Secondary antibodies are: goat anti-rabbit 800CW (#926-32211, LI-COR Biosciences, 1:10000), goat anti-mouse 800CW (#926- 32210, LI-COR Biosciences, 1:10000), anti-rat 680 (#925-68076, LI-COR Biosciences, 1:10000), donkey anti-rabbit 680LT (#926-68023, LI-COR Biosciences, 1:10000), and donkey anti-mouse 680LT (#926-68022, LI-COR Biosciences, 1:10000).

Histone extraction

For histone extraction cells were plated in 10cm dishes and treated with DMSO or 300 nM osimertinib for indicated duration. Cells were harvested by trypsination, washed twice in cold PBS (Sigma-Aldrich, USA) and lysed in PBS containing 0.5 % Triton X 100 (Roth, Germany) for 10min on ice. Samples were then centrifuged (10min, 2000 rpm, 4°C) and washed 1x with PBS+0.5%Triton X 100. Pellets were resuspended in 0.2 N HCl at a density of $4x10^7$ cells/ml and rotated overnight (4°C). Upon centrifugation (10 min, 2000 rpm, 4°C) protein concentration in supernatant was measured using Pierce BCA Protein assay kit (Thermo Scientific, USA). Following addition of 3x Laemmli buffer the pH was normalized using 2 M Tris before immunoblotting as described above.

IVT4 synthesis

RIG-I agonist IVT4 [21,61] (3p-dsRNA) and negative control IVT-GAC (3p-ssRNA) were prepared by in-vitro-transcription from synthetic dsDNA templates

IVT4:

TTGTAATACGACTCACTATA**G**GGACGCTGACCCAGAAGATCTACTAGAAATAGTAGATCTTCTGG GTCAGCGTCCC;

IVT-GAC:

TTGTAATACGACTCACTATA**G**GGAGCGCAGACGCGAGCGCGGCACGGCCGCCAAGGCGAGAC;

T7 promoter underlined, transcription start bold) with T7 RNA polymerase. For transcription of IVT-GAC, no UTP was included in the reaction, preventing non-specific generation of RIG-I ligands.[21] DNA-Template was removed with Turbo DNAse (Thermo Fisher Scientific, Darmstadt. Germany) and RNA was purified by sequential Trizol LS extraction (Thermo Fisher Scientific) and Size-exclusion chromatography with Sephadex G-25 columns (GE Healthcare, Freiburg im Breisgau, Germany). Purity was analyzed by TBE-urea-page, activity and specificity was confirmed by stimulation of human PBMC and THP-1 WT and RIG-I -/-cells.

HLA, B2M, VTCN1 and PD-L1 surface expression via flow cytometry

Cells were harvested, washed with PBS (Sigma Aldrich, USA) and re-suspended in FACS buffer (2% BSA, 2 mM EDTA in PBS). 50,000-200,000 cells were stained with the appropriate amount of antibody for 30 min on ice in the dark. The washed cells were directly analyzed on the Beckman Coulter Gallios 10/3 Flow Cytometer (Beckmann Coulter, USA). Data was analyzed with Kaluza Analysis Software Version 2.1 (Beckman Coulter, USA) and expression was quantified as the mean fluorescence intensity (MFI). Antibodies used: B7-H4/VTCN1 (PE, #358104, BioLegend, 1:40), beta-2 microglobulin (B_2M) (PE, #A15770, ThermoFisher Scientific, 1:100), HLA-ABC (FITC #11-9983-42, ThermoFisher Scientific, 1:40), PD-L1 (CD274, B7-H1 PE-Vio770, #130-122-818, 1:50).

Determination of cytokine concentration in cell culture supernatant

TNF-α, IL-6, IP-10 (BD Biosciences) and IFN-α (eBioScience) secretion of cell lines were measured by ELISA according to the manufacturer's instructions. For quantification of human type I IFN, cell supernatant was incubated with THP1-Dual reporter cells (InvivoGen) for 20 h. THP1-Dual supernatant containing secreted Lucia luciferase was harvested and mixed with Coelenterazine substrate (Synchem). Luminescence was measured and compared with that of a recombinant IFNα2a standard (Miltenyi Biotec) to determine the concentration of type I IFN in the lung cell supernatant.

Nucleic acid receptor (NAR) stimulation

Cell lines were seeded at 2-3x10^4/well in 96-well plates and allowed to adhere overnight. The next day, cells were transfected with 1 µg/ml IVT4, plasmid DNA (pDNA) using Lipofectamine 2000 (Thermo Fisher Scientific), or with 1 µg/ml poly(I:C) using the transfection reagent TransIT-LT1 (Mirus Bio). For stimulation of TLRs, 1 µg/ml R848 or 10 µg/ml poly(I:C) were added to the medium of the cells. After incubation for 16 h, supernatant was harvested and used for cytokine determination.

MTT assay

After NAR stimulation of cell lines for 16 h, metabolic activity as a measure of cell viability was assessed. 20 µl thiazolyl blue tetrazolium bromide (Sigma-Aldrich) solution (5 mg/ml in PBS) were added to each 96-well containing 80 µl medium. After incubation for 40 min at 37°C, the reaction was stopped with 100 µl 10% SDS. The plate was incubated overnight at 37°C to allow dissolving of the formed crystals. Absorption at 570 nm was measured the following day.

***BRAF^{V600E}* overexpression**

Retroviral particles were generated by co-transfecting pBabe-puro empty vector or pBabe-puro carrying *BRAF^{V600E}* ORF with pCL-ampho packaging plasmid using Lipofectamine 2000 (Invitrogen, USA) in HEK293T cells. Virus supernatants were harvested after 48 h, filtered and added to PC9 cells with polybrene (10 µg/ml). Following puromycin selection, PC9-*BRAF^{V600E}* cells were cultured in 100 nM osimertinib. Presence of the mutation was validated by Sanger sequencing and its expression by immunoblot with a mutation specific antibody.

Cell count

For each replicate of the cell counting experiment 100,000 cells per well were seeded as triplicates per timepoint in 12 well plates and left to adhere overnight. Cells were treated with 300 nM osimertinib. The number of cells per well was counted using a Z2 Coulter particle counter (Beckman Coulter) at the treatment start and after indicated treatment duration.

Cell cycle analysis by flow cytometry

For cell cycle analysis, cells were treated with 300 nM osimertinib, 1 µM vemurafenib, 1 µM ceritinib, 100 nM capmatinib, 100 nM neratinib for indicated durations, harvested using trypsin, washed twice in PBS and fixed in ice-cold 70% ethanol overnight. Cells were washed twice in PBS and treated with 100 µg/mL RNase for 10 min at 37 °C. Then cells were stained with 25 µg/mL propidium iodide for 1 h at 37 °C and washed twice with PBS. DNA content was analyzed on a BD Gallios 10/3 flow cytometer (Beckman Coulter, USA) and cell cycle analysis excluding cell doublets was performed using Kaluza software (Beckman Coulter, USA).

Caspase inhibitor analysis

To inhibit caspase-dependent cell death, cells were treated with DMSO or osimertinib (300 nM) +/- Caspase 3/7 inhibitor Z-DEVD-FMK (50µM, Selleckchem, USA) or pan caspase inhibitors Z-VAD-FMK (20 µM, Enzo Lifescience, USA) Q-VD-Oph (10 µM, Selleckchem, USA) for 48-72 hrs. Cell death was quantified as cells positive for propidium iodide (PI) uptake (1 µg/ml) by flow cytometry using an LSR-FACS Fortessa (BD Bioscience) or gene expression was analyzed by RT-qPCR as described below.

Cell death inhibitor analysis

To inhibit cell death pathways following IVT4 treatment, cells were treated with IVT4 (1 ng/µL) as described above in combination with DMSO, pan-caspase inhibitor Z-VAD-FMK (20 µM, Enzo Lifescience, USA), necroptosis inhibitor Nec1s (10µM, Abcam, USA) and ferroptosis inhibitor Fer1 (5 µM, Cayman chemicals) for 24h. Cell death was quantified as cells positive for propidium iodide (PI) uptake (1 µg/ml) by flow cytometry using BD FACScan flow cytometer (Beckman Coulter, USA).

β-galactosidase staining

For ß-galactosidase staining cells were seeded in 6 well plates and left to adhere overnight. Cells were treated with 300 nM osimertinib, 1 µM vemurafenib, 1 µM ceritinib, 100 nM capmatinib, 100 nM neratinib for 5 days and ß-galactosidase activity was measured using the Senescence β-Galactosidase Staining Kit (Cell Signaling Technology, #9860) according to the manufacturer's instructions. Images were taken using the X70 Fluorescence Microscope (Olympus) and the DP74 camera (Olympus). The scalebar indicates 100 µm.

Apoptosis analysis by flow cytometry

For cell death analysis PC9 CRISPRv2 e.v., PC9 sgMAVS or PC9 sgIRF1 cells were plated in 6-wells and left to adhere overnight. Medium was replaced and IVT4 or unspecific control IVT-GAC were mixed with Lipofectamine 2000 (Invitrogen, USA) in Opti-MEM (ThermoFisher, USA) and added to achieve a final concentration of 1ng/µL and 300 nM osimertinib or 0.1 % DMSO as indicated. After 24 h cells and supernatant were harvested and washed twice with PBS (Sigma-Aldrich, USA). Samples were then resuspended in 100 µL 1x binding buffer (70 % PBS with 20 % Accutase (Sigma-Aldrich, USA) and 10x binding buffer (0.1 M Hepes, 1.4 M NaCL, 25 mM $CaCl_2$) with addition of 3 µL PI (0.5 mg/ml) and 2 µL FITC-Annexin V (BD Pharmingen). Samples were incubated 20 min in the dark and subsequently diluted with 1x binding buffer prior to analysis using a FACS Gallios Flow Cytometer and the corresponding FACS Kaluza analysis software (Beckman Coulter, USA). Colo205, A549 and A375 were seeded in 6-wells, left to adhere overnight and were subsequently treated with 100 nM trametinib or 1 µM vemurafenib (only Colo205, A375). After 48 h IVT4 or IVT-GAC were transfected for 24h before measuring cell death via Annexin V/PI flow cytometry as described above.

***NOXA* and *PUMA* knock-down**

PC9 cells were seeded in 6-well plates and left to adhere overnight. The next day siRNA for *NOXA* (SMARTpool L-005275-00-0005, Dharmacon) or *PUMA* (SMARTpool L-004380-00-0005, Dharmacon) or a combination of both were mixed with DharmaFECT-I (T-2001-01, Dharmacon, USA) according to manufacturer's instruction and applied to the cells at a final concentration of 25 nM. After 48h cells were treated with osimertinib (300 nM) + IVT4 (1 ng/µL) for 24h as described above. Cell death was measured using PI staining and flow cytometry as described above.

CRISPR/Cas9-mediated knock out in human cell lines

For gene knock-outs sgRNAs targeting *MAVS*, *STING*, *IRF1* or *IRFNA1* were cloned into lentiCRISPR v2 vector as previously described.[62] lentiCRISPR v2 was a gift from Feng Zhang (Addgene plasmid # 52961). Sequences can be found in **Table S2**. For lentiviral production, HEK293T cells were transduced using lentiCRISPRv2 empty vector (e.v.) or carrying an sgRNA (sgMAVS, sgSTING or sgIRF1) with psPAX2 and pMD2.G helper plasmids using TransIT-LT1 (Mirus Bio, USA) or Lipofectamine 2000 (Invitrogen, USA). psPAX2 and pMD2.G were a gift from Didier Trono (Addgene plasmids #12260 and #12259). 48 hr after transfection the supernatant of transduced HEK293T cells was collected and added to the target cells (PC9) in presence of 10 µg/ml polybrene (Santa Cruz Biotechnology). Puromycin (1µg/ml) selection was started the next day and continued until all non-transfected control cells treated in parallel were killed. Knock out efficacy was confirmed using immunoblot in cell lines with detectable protein expression. For validation of STING-knock out in PC9 cells, amplicons of the STING gene were PCR amplified from PC9 e.v. and PC9 sgSTING. Ampure XP bead-purified PCR-amplicons, 500ng each, were prepped using the Illumina Nextera DNA Flex protocol (Illumina, USA) with 5 cycles of PCR and the post-PCR clean-up. After validation (2200 TapeStation; Agilent Technologies) and quantification (Qubit System; Invitrogen, USA), amplicon libraries were individually quantified using the KAPA Library Quantification kit (Peqlab, Germany) and the 7900HT Sequence Detection System (Applied Biosystems, USA) and subsequently spiked-in in larger pools of libraries. The pools were sequenced on an Illumina NovaSeq6000 (Illumina, USA) using a paired-end 2×150 bp protocol. Sequencing reads were aligned to the human reference genome GRCh38 with Bowtie2 [63], efficacy of genome editing was inspected using the Integrated Genome Viewer (IGV v2.3.68).

CRISPR/Cas9-mediated generation of PC9$^{T790M+C797S}$

For CRISPR/Cas9-based genome editing 500,000 PC9 cells were plated in a 6-well and electroporated with Cas9 RNP electroporation mix (300 nM Cas9, 25 nM EGFR sgRNA, 200 pmol ssDNA repair template) in 140 µl Opti-MEM (Life Technologies) using a NEON electroporator (Life Technologies) (protocol: 1230 V, 30 ms, 2 pulses). After 24 h PC9 bulk cells cells were selected with 300 nM osimertinib for 5 days. Successful gene editing was

confirmed via Sanger sequencing of *EGFR* Exon 20 and with deep sequencing (amplicon size <150 bp).

single cell (sc)RNA-seq analysis

For scRNA library construction, we made use of the Chromium™ Single Cell 3' Reagent Kits v2 and the Chromium controller (10x Genomics, USA). PC9 or Colo205 control cells (0h) or treated for 24h and 72h with 300nM osimertinib or 1µM vemurafenib, respectively, were suspended as single cells in 1X PBS containing 0,04% BSA and checked for viability (> 75%), debris or cell aggregates. Cells are delivered at a limiting dilution, such that the majority (~90- 99%) of generated gel in emulsion beads (GEMs) contains no cell, while the remainder largely contain a single cell. 2.000 cells per sample were targeted. Upon dissolution of the Single Cell 3' Gel Bead in a GEM, primers containing (i) an Illumina R1 sequence (read 1 sequencing primer), (ii) a 16 bp 10x Barcode, (iii) a 12 bp Unique Molecular Identifier (UMI) and (iv) a poly-dT primer sequence are released and mixed with cell lysate and Master Mix. Incubation of the GEMs then produces barcoded, full-length cDNA from poly-adenylated mRNA. Subsequently GEMs are broken, the pooled fractions are recovered and silane magnetic beads are used to remove leftover biochemical reagents and primers from the mixture. Full-length, barcoded cDNA is then amplified by PCR to generate sufficient mass for library construction. Enzymatic Fragmentation and Size Selection are used to optimize the cDNA amplicon size prior to library construction. R1 (read 1 primer sequence) are added to the molecules during GEM incubation. P5, P7, a sample index and R2 (read 2 primer sequence) are added during library construction via End Repair, A-tailing, Adaptor Ligation and PCR. The final libraries contain the P5 and P7 primers used in Illumina bridge amplification. A Single Cell 3' Library comprises standard Illumina paired-end constructs which begin and end with P5 and P7. We pooled the libraries and allocated 1 lane of an Ilumina HiSeq4000 flowcell (Illumina, USA) aiming at >50k reads/cell. Resulting FASTQ-files were processed using the Cellranger Pipeline v2.0.1 (10x Genomics, USA) including alignment to hg19. Filtered gene barcode matrices were further analyzed using the R package Seurat v3.0.2 [64]. Cells with < 10% mitochondrial reads, with at least 200 detected genes and unique molecular identifier (UMI) counts within two standard deviations of the mean per time point were retained before samples were merged and normalized using SCTransform. Subsequently PCA was performed on genes of the adult tissue stem cell (ATSC) signature of the normalized data and cells clustered using the uniform manifold

approximation and projection (UMAP) dimension reduction technique[65]. Relative expression of selected genes was visualized per cell on the sctransform embedding and as heatmaps.

Pseudotime analysis of control PC9 cells was performed using Monocle3 v0.1.2[66,67]. The filtered gene barcode matrix imported into R with Seurat v3.0.2 [64]. Cells with < 10% mitochondrial reads, with at least 200 detected genes and unique molecular identifier (UMI) counts within two standard deviations of the population mean were retained for further analysis. Signatures containing genes specific for G1/S or G2/M were obtained from Tirosh et al [15,16,24,32,48], however Cyclin E and B were removed. Genes of these gene sets were subsequently used to cluster cells along a pseudo-time trajectory by performing data normalization, PCA and UMAP embedding with Monocle3. Expression of genes known to be differentially regulated with cell cycle progression including cyclins was used to validate the ordering of cells by cell cycle phases before genes of interested were investigated for cell cycle dependent expression variation.

Treatment of the humanized mouse model

In the pilot experiment, osimertinib was given daily for 4 days at a dose of 5mg/kg mouse weight by oral gavage. Control animals were treated with vehicle (1 % DMSO, 30 % PEG300, 69 % H_2O). At the end of the experiment, tumors were harvested, halved and one part immediately frozen in liquid nitrogen and the other part fixed in 4% formalin in PBS for 24 h at 4 °C. Infiltration with lymphocytes was assessed by IHC as described below. In a second experiment, treatment was initiated with osimertinib or vehicle was performed for 4d described above. After discontinuation of osimertinib, 100µg Nivolumab (Bristol-Myers-Squibb) or vehicle (PBS) was given 2x/week i.p. Tumor growth was monitored as shown in the respective figures (at least 2x/week) by caliper measurement. At the end of the experiment, tumors were harvested and frozen in liquid nitrogen. In a third experiment, once treatment commenced (day 17, average tumor size approximately 150 mm^3), osimertinib or vehicle was given for 4 days as described above followed by intratumoral IVT-4 or control IVT4-GAC administration 2/weekly. Briefly, 20 µg IVT4 or IVT4-GAC was mixed with 10% glucose in sterile H_2O for a total volume of 12.5µl. Sterile water was added for a total volume of 25 µl followed by vortexing. Separately, 3µl in vivo-jetPEI (Polyplus, France) was added to 9.5 µl of 10 % glucose. 12.5 µl sterile H2O was added followed by vortexing. Next, both tubes were combined, vortexed and incubated for 15 min at RT. Finally,

50 µl of the resulting mix was injected into each tumor. At the end of the experiment, tumors were harvested and immediately frozen in liquid nitrogen.

Flow cytometry of humanized mice

For Flow cytometry analyses humanized mice were treated for four days with osimertinib or vehicle p.o before sacrifice or with a 4d lead-in of osimertinib or vehicle followed by IVT4/IVT-GAC as described above for 6d. S.c. tumors have were harvested and processed according to [22,68]. The following antibodies (BioLegend, San Diego, CA, US) were used: CD3 (PE, UCHT1, 1:100), CD4 (APC, RPA-T4, 1:100), CD45 (PE/Dazzle™594, HI30, 1:100), CD69 (APC/Cy7, FN50, 1:100), CD8 (Alexa Fluor®700, SK1, 1:100), PD-1 (Pacific Blue, EH12.2H7, 1:100), PD-L1 (PE/Cy7, 29E.2.A3, 1:100), and TIM-3 (FITC, F38-2E2, 1:100). Flow cytometry was performed on a Gallios 10/3 and data was analyzed using Kaluza 2.1 (Beckman Coulter, Brea, CA, US).

IHC of humanized mice

Mouse tumors of the pilot study were formalin-fixed and paraffin embedded (FFPE). For IHC 2 µm thick sections were cut from FFPE tissue blocks and stained according to standard procedures on a Leica Bond MaxTM system (Leica Microsystems, Wetzlar, Germany) using monoclonal primary antibodies directed against CD3 (clone SP7, 1:50, Thermo Fisher Scientific, MA, USA), CD4 (clone 4B12, 1:100, Thermo Fisher Scientific, MA, USA) and CD8 (clone C8/144B, 1:200,DAKO Glostrup Denmark). Manual quantification of lymphocyte infiltration was performed in a blinded fashion by a trained pathologist (P.L.) as low (-), medium (+) or high (++).

Artificial Intelligence-based IHC analysis

Tissue sections were scanned at 40X using a Hamamatsu NanoZoomer S360 digital whole-slide image (WSI) scanner. For the purpose of tumor segmentation on WSIs, a U-net deep-convolutional neural network was trained on a NVIDIA Quadro RTX 6000 GPU (NVIDIA, USA) using Adam as optimizer. For generalizability, different staining's were included within the training data. For quantification purpose, a watershed algorithm for cell detection was applied, while results were checked for plausibility by a trained pathologist as an observer.

Imaging mass cytometry

Imaging mass cytometry (IMC) was carried out using the Hyperion Imaging System (Fluidigm). FFPE sections were prepared at 3 µm thickness and immediately processed according to the manufacturer's protocol. Deparaffination, rehydration, antigen retrieval and blocking steps were conducted by an automated staining system (Ventana BenchMark Ultra, Roche). The antibody- cocktail (Immune-Oncology Panel Kit, Fluidigm) was prepared on ice. Antibodies were diluted in Bond wash solution (Leica Biosystems). Every slide was incubated with the antibody cocktail for 4 hours at room temperature in a hydration chamber followed by an overnight incubation at 4°C. Afterwards, DNA-counterstaining was done using an intercalating DNA dye following the standard protocol. Finally, the slides were air-dried and kept in a dry box until imaging. Images were acquired with the Hyperion Imaging System coupled to a Helios Mass Cytometer (Fluidigm). Haematoxylin and eosin-stained slides were used to guide the selection of regions of interest (ROIs). ROIs were scanned by a pulsed UV laser where each pulse sampled the stained tissue to detect by CYTOF metal labeled antibodies per coordinate. All images were acquired at 200 Hz, laser diameter 1µm. Of the stained antibodies images for CD3 (17Er, dilution 1:50), CD8A (162Dy, 1:100), Pan-cytokeratin (CK, 148Nd, 1:400), Granzyme B (167Er, 1:50), Ki67 (168Er, 1:100) and nucleic acid dye (191Ir/193Ir, 1:500) were generated using the MCD Viewer software (Fluidigm).

A549 xenograft mouse model

For A549 xenograft mouse models 6-8 weeks old female NPSG mice were inoculated with $1x10^6$ A549 cells s.c. per flank. Tumor volume was subsequently measured once a week and mice randomized into treatment groups when tumor volumes reached 150-200mm^3. Treatment with trametinib (3mg/kg/d) or vehicle was given p.o. from day 0 until end of the study. IVT4 or control IVT-GAC was administered for 14 days starting on study day 5 as i.t. injections (20µg per tumor, complexed with in vivo-JetPEI, Polyplus, France) twice per week. Tumor volumes and body weight were measured regularly during the study.

Treatment of the syngeneic *Egfr^{mut}* lung cancer mouse models

In a first cohort, n=8 mice were randomized to receive 10d vehicle or 0d, 4d or 10d osimertinib (5mg/kg, 5 days/week, n=2 per group) before mice were sacrificed. One tumor

per mouse was analyzed by RNA-seq. To test the effects of IVT4 with osimertinib, a second cohort was subsequently inoculated. When the average tumor volume reached approximately 200 mm^3, the mice were randomly assigned to groups (3 mice/group, two tumor per mouse) that were first treated for 7d with vehicle or osimertinib (5 mg/kg/day, 5 days/week, p.o.) before IVT4 or IVT-GAC (20 µg/tumor/day, 2 days/week, tumor injection as described above) were added for a total duration of 28 days. Tumor volume ($width^2$ ×length/2) was determined periodically.

For the immune cell depletion cohorts, mice were inoculated and treated with osimertinib/vehicle and IVT4/IVT-GAC as described above. In addition, i.p. injections with either InVivoMab anti-NK1.1 antibody (300µg/injection; clone PK136, BioXcell, USA), anti-mouse CD8 (250µg/injection; clone 53-6.7, BioLegend, USA) or IgG2a isotype control (250µg/injection) were administered 3 and 6 days before start of IVT4/IVT-GAC and then once weekly until the end of the study. Depletion efficacy was assessed by FACS analysis of splenic cell type composition after the study.

Lymphocyte analysis by flow cytometry in syngeneic *Egfr^{mut}* mouse model

To assess lymphocyte composition in tumors or spleens, tissues of treated mice were dissociated into single-cell suspensions by using Tumor Dissociation Kit, mouse (#130-096-730, Miltenyi Biotec) and/or a 70µm cell strainer (CORNING, USA). Then cells were stained with indicated fluorescence-labeled antibodies and subjected to Flow cytometric analysis. Cells were washed and incubated with mAbs for 30 min at 4 °C. The following mAbs were used: anti-mouse CD3 (clone 17A2, BioLegend, USA, 1:50), anti-mouse CD4 (clone GK1.5, eBioscience, USA), anti-mouse CD8 (clone 53-6.7, eBioscience, USA, 1:50), anti-mouse Foxp3 (clone FJK-16s, eBioscience, 1:50), anti-mouse NK1.1 (clone PK136, eBioscience, USA, 1:50) and anti-mouse CD49b (clone DX5, BioLegend, USA). We determined a suitable gate for lymphocytes (TILs), compared with that of spleen cells from untreated mice. For analysis of tumor-infiltrating lymphocytes mice were inoculated and treated with osimertinib lead-in followed by injection of IVT4/IVT-GAC for 10d. At harvest tumors were dissociated into single cells and stained using the following antibodies: CD3 (17A2, #46-0032-82; #100222, 1:50), CD8 (53-6.7, BioLegend #100714), PD-1 (29F.1A12, #135210, 1:50), Zombie Dye (#423133, 1:50), CD45 (30-F11, #103108, 1:50), NK1.1 (PK136, #25-5941-82, 1:50).

Samples were analyzed using a MACSQuant flow cytometer (Miltenyi Biotec), and data were analyzed using FlowJo software version 10.2 (TreeStar, Ashland, OR, USA).

EGFR^{mut} PDX models

Tumor fragments of derived from two patients with EGFR mutation positive lung cancer (LU1235, LU6422) were propagated in female BALB/c nude mice (Crown Bioscience Inc.). The protocol and any amendments or procedures involving the care and use of animals in this study was reviewed and approved by the Institutional Animal Care and Use Committee (IACUC) of CrownBio prior to execution. During the study, the care and use of animals were conducted in accordance with the regulations of the Association for Assessment and Accreditation of Laboratory Animal Care (AAALAC). Mice 6-8 weeks old were randomly enrolled into vehicle (1% DMSO and 30% PEG300 in H_2O)– or osimertinib (5 mg/kg)–treatment groups (oral gavage) when the average tumor volume reached approximately 400mm^3. Tumor volume was measured twice weekly using a caliper and the tumor volume was calculated as $V = (L\text{x}W\text{x}W)/2$, where L and W represent tumor length and width, respectively. In the vehicle group mice were sacrificed on d0 or d7 of the treatment phase (each n=2), osimertinib treated mice were sacrificed on d1 or d7, 24h after the first or last dose, respectively (each n=2). Tumors were snap frozen and subjected to RNA-seq analysis as described above.

RT-qPCR

Total RNA was isolated using the RNeasy Mini kit (#74101 Qiagen, Germany) according to the manufacturer's instructions including DNAse digestion. In all, 1 µg of total RNA was reverse transcribed using HiScript II 1st Strand cDNA Synthesis Kit (#R211, Vazyme, China) using random hexamere primers. Quantitative real-time PCR (qPCR) was performed using 7900HT Real-Time PCR System (Applied Biosystems) with the ChamQ Universal SYBR qPCR Master Mix (#Q711, Vazyme, China). qPCR primers used to analyze mRNA levels are listed in Supplementary Table S2. Data were normalized to expression of GAPDH or β-Actin and difference of treated vs. untreated cells calculated as ddCt. For visualization fold-changes were calculated as 2^ddCt, significance between cell lines was assessed based on log fold-changes.

IRF1 overexpression

For transient IRF1 overexpression cells were seeded in 6-well plates or 6 cm dishes and left to adhere over night. pcDNA3.1-empty vector or pcDNA3.1-IRF1 (Genscript, USA) was then mixed in Opt-MEM with TransIT-LT1 (Mirus, USA) and added to the cells. After 72h expression was analyzed using immunoblot or RT-qPCR as described above. For cell death analysis in PC9 cells, 48h after IRF1 transfection the medium was changed and IVT4/IVT-GAC was transfected for 24h prior to AnnexinV/PI analysis as described above.

RNA-sequencing of human cell lines and *EGFR^mut^* mouse model samples

For RNA extraction from cell lines, cells were harvested by trypsination and resuspended in RLT buffer (#74104 Qiagen, Germany) supplemented with β-mercaptoethanol (Roth, Germany). Total RNA was subsequently extracted using the RNeasy Mini Kit (#74104 Qiagen, Germany). For extraction of tumor RNA, tumor area was circled by a pathologist on HE stained slides. 5-10μm thick section of the fresh-frozen tissue specimens were cut before RNA was extracted from to the indicated tumor containing areas with the RNAeasy Mini kit (#74104 Qiagen, Hilden). 3´ UTR mRNA libraries were generated from total RNA using the Lexogen QuantSeq kit (Lexogen, Austria) according to the standard protocol. After validation (2200 TapeStation; Agilent Technologies) and quantification (Qubit System; Invitrogen, USA), pools of cDNA libraries were generated. The pools were quantified using the KAPA Library Quantification kit (Peqlab, Germany) and the 7900HT Sequence Detection System (Applied Biosystems, USA) and subsequently sequenced on an Illumina HiSeq4000 or a NovaSeq sequencer (Illumina, USA) using a 1x50bp or 1x75 bp protocol.

RNA-seq analysis of human cell lines and *EGFR^mut^* mouse model samples

Sequencing data were processed as described previously [29,69]. FASTQ files containing adapter-trimmed reads were mapped to the human reference genome GRCh38 or the mouse reference genome GRCm38, respectively using the STAR aligner[49,50,70] and expression quantification with RSEM[24,51,71]. Differential gene expression and adjusted expression fold-changes between controls and treated samples were calculated from expression counts using DESeq2[1-3,72]. In differential expression models variables like cell line, batch or treatment time-point were included as co-variates if appropriate. Resulting p-values were adjusted using Benjamini-Hochberg correction.[73] Enrichment of biological functions among differentially expressed genes was assessed with gene set enrichment analysis (GSEA)[74,75] with gene sets of the MSigDB Hallmark collection [76] to which we added the senescence-

associated “adult tissue stem cell” (ATSC) gene set[27,28]. To capture magnitude, direction and variation of gene expression, genes were ranked based on the differential expression test statistic prior to unweighted GSEA with the preranked tool. q-values were derived from FDR-corrected p-values. For murine samples, murine genes were mapped to human orthologs using the R package biomaRt [77] prior to GSEA analysis.

RNA-seq data of *BRAF*^{mut} melanoma patients[34] were obtained from the European Genome Archive (EGAS00001000992) and processed as described above. Immune cell subtype abundances were estimated using transcripts per million (TPM) of melanoma patient RNA-seq with CIBERSORT (LM22 reference cell matrix, 500 permutations, no quantile normalization, final estimation p-value < 0.1)[35,78] For expression analysis of TCGA lung adenocarcinoma (LUAD) patients, RNA-seq V2 Level3 RSEM normalized gene expression of the TCGA GDAC Firehose standard run 2016-01-28 (doi:10.7908/C11G0KM9) was downloaded from the GDAC website (http://gdac.broadinstitute.org) and used as input for CIBERSORT (LM22 reference matrix, 1000 permutations, no quantile normalization, relative mode). Patients with an estimation p-value < 0.05 were included in downstream analyses (n=350). For binned data plots, patients were grouped into equally sized bins based on *DUSP6*/*SPRY4* expression and median expression and CD8 T-cell proportion per bin were calculated prior to correlation analysis. Spearman correlation coefficients of CD8 T cell proportion with the expanded set of MAPK activity genes [45] was calculated on unbinned patient data.

For single sample gene set enrichment analysis (ssGSEA) the R package GSVA[79] was used to assess enrichment of hallmark E2F, MYC V1, MYC V2, IFN-alpha and IFN-gamma gene sets of the MSigDB collection and the ATSC gene set[27,28,76] in treatment naïve *BRAF*^{mut} melanoma patients (n=14) and TCGA lung adenocarcinoma patients (n=515). Association between gene set were calculated across patients using Pearson correlation of enrichment scores.

RNA-sequencing of primary murine PDAC cell lines

Four cell lines derived from different *Ptf1a*$^{wt/Cre}$; *Kras*$^{wt/LSL\text{-}G12D}$; *Trp53*$^{loxP/loxP}$ mice were treated for 48 h with their individual GI_{50} concentrations of trametinib. RNA was isolated using Maxwell® RSC simplyRNA Cells Kit (Promega, USA) according to manufacturer’s instruction. Sequencing was performed by the CeGaT GmbH (Tübingen, Germany) with

100 ng input RNA for library preparation with the TruSeq Stranded mRNA Kit (Illumina, USA). Afterwards, 2 x 100 bp were sequenced on a HiSeq 4000 (Illumina, USA). For quantification, salmon v. 0.12 [80], TXImport v. 1.6 and DESeq2 v. 1.18 [72] were used.

ChIP-analysis

ChIP-analyses were performed as described previously.[69] Briefly, PC9 cells were plated in 15cm dishes and left to adhere overnight before treatment with 300nM osimertinib or DMSO control treatment was started. After Briefly, 1*10^7 cells were cross-linked with 1 % formaldehyde, lysed and chromatin was sheared. DNA-Protein complexes were immunoprecipitated with specific antibodies against RNA-Pol2 (CST#14958), RNA-Pol2 CTD pSer5 (CST#13523), RNA-Pol2 CTD pSer2,5 (CST#13546), or unspecific mouse IgG (sc-2025, Santa Cruz Biotechnology) and captured using protein G Dynabeads (Thermo Fisher Scientific). After washing, eulution and decrosslinking, ChIP DNA was extracted using the ChIP DNA Clean & Concentrator Kit (Zymo Research) and analyzed by qRT-PCR using primers directed against transcription start site, gene body, and transcription termination regions of genes *IRF1*, *VTCN1*, and *DUSP6*. The primers are listed in Supplementary Table 2. ChIP signal was calculated as percentage of input DNA.

Supplementary References

61. Goldbeck, M., Schlee, M., Hartmann, G. & Hornung, V. Enzymatic Synthesis and Purification of a Defined RIG-I Ligand. in *Innate DNA and RNA Recognition: Methods and Protocols* (eds Anders, H. J. & Migliorini, A.) 15-25 (Springer 2014).
62. Sanjana, N. E., Shalem, O. & Zhang, F. Improved vectors and genome-wide libraries for CRISPR screening. *Nat Methods* **11,** 783–784 (2014).
63. Langmead, B. & Salzberg, S. L. Fast gapped-read alignment with Bowtie 2. *Nat Methods* **9,** 357–359 (2012).
64. Stuart, T. *et al.* Comprehensive Integration of Single-Cell Data. *Cell* **177,** 1888–1902.e21 (2019).
65. McInnes, L., Healy, J. & Melville, J. UMAP: Uniform Manifold Approximation and Projection for Dimension Reduction. *ArXiv e-prints* 1–51 (2018).
66. Qiu, X. *et al.* reversed graph embedding resolves complex single-cell trajectories. *Nat Methods* 1–10 (2017). doi:10.1038/nmeth.4402
67. Cao, J. *et al.* The single-cell transcriptional landscape of mammalian organogenesis. *Nature* 1–31 (2019). doi:10.1038/s41586-019-0969-x
68. Meder, L. *et al.* Combined VEGF and PD-L1 Blockade Displays Synergistic Treatment Effects in an Autochthonous Mouse Model of Small Cell Lung Cancer. *Cancer Research* **78,** 4270–4281 (2018).

69. Brägelmann, J. *et al.* Systematic Kinase Inhibitor Profiling Identifies CDK9 as a Synthetic Lethal Target in NUT Midline Carcinoma. *CellReports* **20,** 2833–2845 (2017).
70. Dobin, A. *et al.* STAR: ultrafast universal RNA-seq aligner. *Bioinformatics* **29,** 15–21 (2013).
71. Li, B. & Dewey, C. N. RSEM: accurate transcript quantification from RNA-Seq data with or without a reference genome. *BMC Bioinformatics* (2011). doi:10.1186/1471-2105-12-323
72. Love, M. I., Huber, W. & Anders, S. Moderated estimation of fold change and dispersion for RNA-seq data with DESeq2. *Genome Biol* **15,** 31 (2014).
73. Benjamini, Y. & Hochberg, Y. On the adaptive control of the false discovery rate in multiple testing with independent statistics. *J. Educ. and Behav. Statistics* **25**, 60-83 (2000).
74. Mootha, V. K. *et al.* PGC-1alpha-responsive genes involved in oxidative phosphorylation are coordinately downregulated in human diabetes. *Nat Genet* **34,** 267–273 (2003).
75. Subramanian, A. *et al.* Gene set enrichment analysis: a knowledge-based approach for interpreting genome-wide expression profiles. *Proc Natl Acad Sci USA* **102,** 15545–15550 (2005).
76. Liberzon, A. *et al.* The Molecular Signatures Database Hallmark Gene Set Collection. *Cell Systems* **1,** 417–425 (2015).
77. Durinck, S., Spellman, P. T., Birney, E. & Huber, W. Mapping identifiers for the integration of genomic datasets with the R/Bioconductor package biomaRt. *Nat Protoc* **4,** 1184–1191 (2009).
78. Chen, B., Khodadoust, M. S., Liu, C. L., Newman, A. M. & Alizadeh, A. A. Profiling Tumor Infiltrating Immune Cells with CIBERSORT. *Methods Mol. Biol.* **1711,** 243–259 (2018).
79. Hänzelmann, S., Castelo, R. & Guinney, J. GSVA: gene set variation analysis for microarray and RNA-seq data. *BMC Bioinformatics* **14,** 7 (2013).
80. Patro, R., Duggal, G., Love, M. I., Irizarry, R. A. & Kingsford, C. Salmon provides fast and bias-aware quantification of transcript expression. *Nat Methods* **14,** 417–419 (2017).

Supplementary Table 2. Primers used for RT-qPCR, ChIP-qPCR, CRISPR cloning and targeted sequencing.

**RT-qPCR primers**			
**Gene**	**Forward (5’-3’)**	**Reverse (5’-3’)**	**Source**
**Human**			
DDX58	CCAGCATTACTAGTCAGAAGGAA	CACAGTGCAATCTTGTCATCC	Sheng et al., Cell 2018
IFIH1	GAGCAACTTCTTTCAACCACAG	CACTTCCTTCTGCCAAACTTG	Sheng et al., Cell 2018
IFIT2	AAGAGGAAGATTTCTGAAGAGTGC	TCTCCAAGGAATTCTTATTGTTCTC	Walter et al., Mol Can Res 2017
TLR3	TGGTTGGGCCACCTAGAAGTA	TCTCCATTCCTGGCCTGTG	Sheng et al., Cell 2018
OAS1	GTGTGTGTCCAAGGTGGTAAAG	ATTTAACTGATCCTGAAAAGTGGTG	Walter et al., Mol Can Res 2017
OAS2	GGAGCTTCCTGATTGGCAGA	ATGTAGGGTGGCAAGCACTG	Goel et al., Nature 2017
IFIT1	CTCACATTTGCTTGGTTGTC	CAACCATGAGTACAAATGGTG	Gratia et al., JEM 2019

MX1	GTTTCCGAAGTGGACATCGCA	CTGCACAGGTTGTTCTCAGC	Gratia et al., JEM 2019
IFI44L	AACTGTGGTATAGCATATGTGG	CTCTCAATTGCACCAGTTTCC	Gratia et al., JEM 2019
GBP1	GAAGTGCTAGAAGCCAGTGC	CCACCACCATAGGCTGTGTA	Bai et al., Sci Rep 2018
GAPDH	AGCCACATCGCTCAGACAC	GCCCAATACGACCAAATCC	Coll et al., Cell Stem Cell 2018
IRF1	TTTGTATCGGCCTGTGTGAATG	AAGCATGGCTGGGACATCA	
HLA-B	CAGTTCGTGAGGTTCGACAG	CAGCCGTACATGCTCTGGA	Goel et al., Nature 2017
PMAPI1	AAGTTTCTGCCGGAAGTTCA	GCAAGAACGCTCAACCGAG	
BBC3	GACCTCAACGCACAGTACGAG	AGGAGTCCCATGATGAGATTGT	
IRF7	CTTCGTGATGCTGCGGGATA	GTGCCTGGGCCTTCTCG	
IRF9	TCCTCCAGAGCCAGACTACT	CGCCCGTTGTAGATGAAGGT	
B2M	GAGGCTATCCAGCGTACTCCA	CGGCAGGCATACTCATCTTTT	Goel et al., Nature 2017
**Murine**			
Actb	AGTGTGACGTTGACATCCGT	GCAGCTCAGTAACAGTCCGC	Liu et al., Science 2019
Irf1	CCCACAGAAGAGCATAGCAC	AGCAGTTCTTTGGGAATAGG	Bonelli et al., EMM 2019
Ddx58	CAGATCCGAGACACTAAAGGGA	TCCTCATCAGCCTTGCTTTCA	
Ifit1	tgttgaagcagaagcacaca	tctacgcgatgtttcctacg	Pichlmeir et al., Nat Imm 2011

sgRNA sequences

Gene	sgRNA target sequence	Source
MAVS	AGTACTTCATTGCGGCACTG	Canadas et al., Nat Med 2018
STING	GGTACCGGGGCAGCTACTGG	Canadas et al., Nat Med 2018
IRF1	TTAATTCCAACCAAATCCCG	DepMap Project (Meyers et al., Nat Gen 2017)
IFNAR1	CGCCACGGCGACGAGCACTA	DepMap Project (Meyers et al., Nat Gen 2017)
EGFR_sgRNA	GAAATTAATACGACTCACTATA GGATAGTCCAGGAGGCAGCCGA GTTTTAGAGCTAGAAATAGC	
EGFR_ssDNA_repair	GAAGCCTACGTGATGGCCAGCGTGGACAACCCCCACGTGTGCCGCCTGCTGGGCATCT GCCTCACCTCCACCGTGCAGCTCATCATGCAGCTCATGCCGTTCGGCAGCCTCCTGGACT ATGTCCGGGAACACAAAGACA	

DNA sequencing primers

Gene	Forward (5'-3')	Reverse (5'-3')
STING_gDNA_1	CATCCATCCATCCCGTGTCC	GATGGCCCACCTGGCA
STING_gDNA_2	CTTGGTTCTGCTGAGTGCC	ATGGCCCACCTGGCA
EGFR_gDNA_Deep_1	CCAGGAAGCCTACGTGATGG	CCAATATTGTCTTTGTGTTCCCGG
EGFR_gDNA_Deep_2	CCCTCCAGGAAGCCTACGT	TTGTCTTTGTGTTCCCGGACA
EGFR_gDNA_Deep_3	AAGCCTACGTGATGGCCAG	GGGAGCCAATATTGTCTTTGTGT
EGFR_gDNA_Exon_20	CCCATTTCCCAGAGCACAGT	CAGACCACACTGAGCACTCA

ChIP-qPCR primers

ChIP-IRF1-TSS	GCACGTCTTGCCTCGACTAA	CTGGGGAATCCCGCTAAGTG
ChIP-IRF1-GB	AAGCCACAGGTCAAGGTTGT	ACATGGTGGCCCATTAGTGC

ChIP-IRF1-TES	TCTGGGACTGAGAAGGTGGAA	TAGGTAGGCTGGCCAAGAGA
ChIP-VTCN1-TSS	GCTCCCGTGTATTGGCCTTA	TTTCCTCATACCTGAGCCGT
ChIP-VTCN1-GB	GGCATTTCCTAATGGGGGCT	GCCCTGTGGAAAAAGTCGTG
ChIP-VTCN1-TES	CCTCTGGGAATTTTCCATTTGGC	TCAGATAAAGACCCAGAGACTCA
ChIP-DUSP6-TSS	TGGATACAAACAGCGAGCGT	CAACACAACCTGTTCCAGCC
ChIP-DUSP6-GB	CCACCTGCCAGAACGAGAAA	GGGGTTCGTAGGATGCTTGT
ChIP-DUSP6-TES	GCGCATGCTAGGGGAAAAAG	TCCAGTCTGTTGTCATGGGC

Supplementary Figures

Supplementary Fig. 1

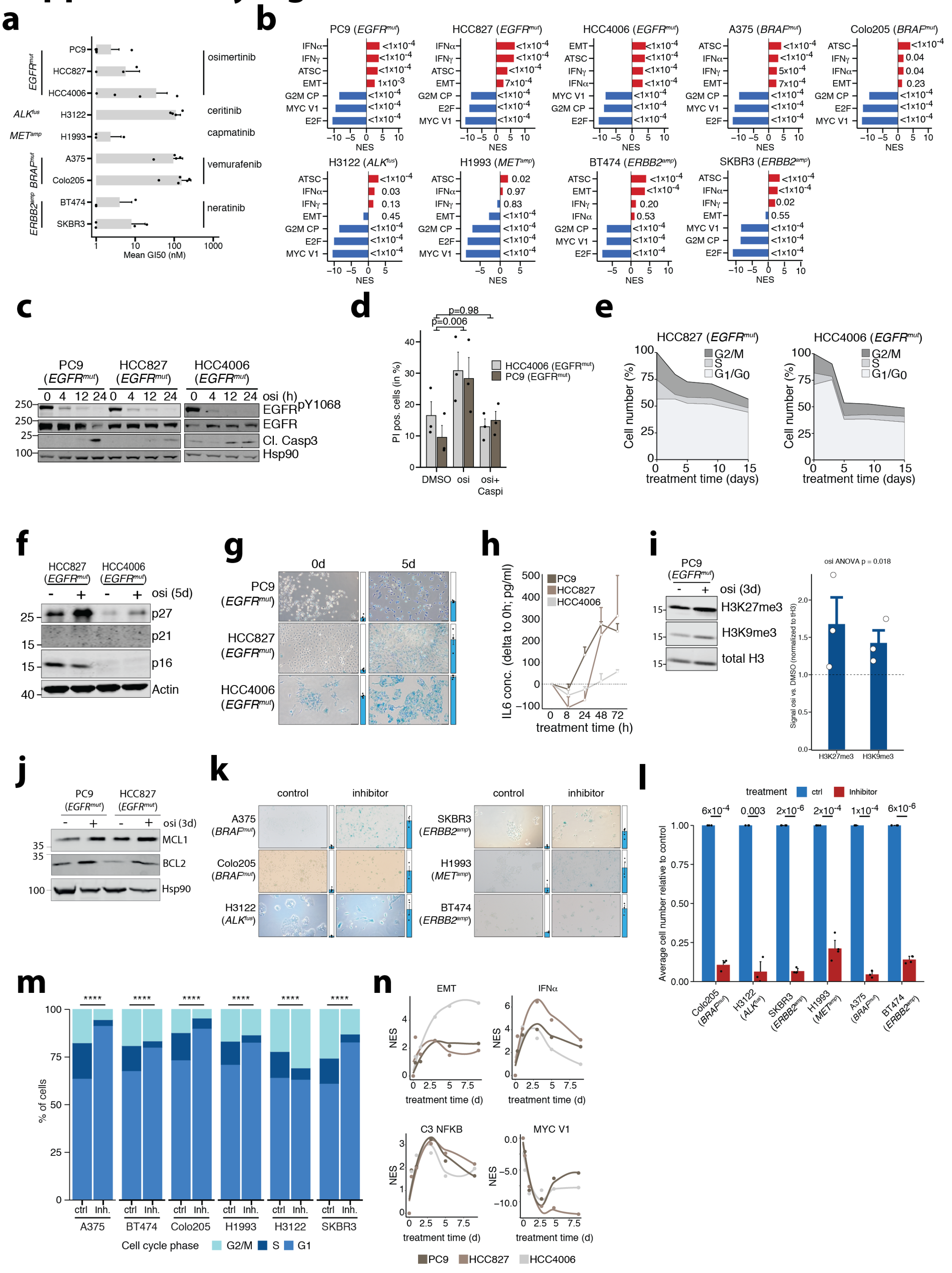

Supplementary Fig. 1: Kinase inhibition induces senescence-associated inflammatory signaling.

(**a**) GI_{50} values of kinase-driven cancer cell lines treated with osimertinib (PC9 n=5, HCC827 n=5, HCC4006 n=4), ceritinib (H3122 n=4), capmatinib (H1993 n=3), vemurafenib (Colo205 n=5, A375 n=4) or neratinib (BT474 n=3, SKBR3 n=4) based on CTG viability assays (mean+SEM of independent replicates indicated by n) (**b**) GSEA for individual cell lines from (a) following RNA-seq after treatment with the respective kinase inhibitors for 72h. (FDR-adjusted q-values by Kolmogorov Smirnov based permutation test) (**c**) Immunoblot of cleaved caspase 3 and EGFR in $EGFR^{mut}$ cell lines treated with osimertinib (osi, 300 nM). Representative image of n=3 independent experiments. (**d**) FACS analysis of caspase dependent cell death induction by combining osimertinib (300 nM) treatment with the caspase inhibitor (Caspi) zVAD (20 µM) followed by propidium iodide (PI) staining. (Mean+SEM of n=3 independent biological replicates) (**e**) Cell count of $EGFR^{mut}$ HCC827 and HCC4006 cells under osimertinib (300 nM) treatment. Upper line indicates average normalized cell number, color shaded areas indicate cell cycle distribution over time. (n=3 independent replicates each) (**f**) Immunoblot of cell cycle regulators p16 (CDKN2A), p27 (CDKN1B) and p21 (CDKN1A) in $EGFR^{mut}$ cells following osimertinib treatment. Representative image of n=3 independent experiments. (**g**) Representative β-galactosidase staining of $EGFR^{mut}$ cell lines treated with osi (300 nM, 5 d) and quantification (mean+SEM of n=4 independent biological replicates). Scale bar 100µM. (**h**) IL6 concentration in cell culture supernatant measured by ELISA during osimertinib treatment. (Mean+SEM of n=3 independent replicates) (**i**) Immunoblot of histone extract of PC9 cells for markers of repressive histone configurations with quantification of Histone mark to total Histone 3 (tH3) per sample in osi vs. DMSO treated cells. (Mean+SEM of n=3 independent experiments) (**j**) Immunoblot of BH3 family members under osimertinib treatment. Representative image of n=3 biological replicates. (**k**) Representative β-galactosidase staining of oncogene-driven cell lines treated with their respective inhibitor (see Fig. S1a, vemurafenib 1µM, neratinib 100nM, ceritinib 1µM, capmatinib 100nM) and quantification (mean+SEM of independent biological replicates n=3 for BT474, H1993 and H3122, n=4 other cells) Scale bar 100µM (**l**) Cell number counts of oncogene-driven cells treated with their respective inhibitor for 5d normalized to controls (ctrl; Mean+SEM of independent biological replicates n=3 H3122, A375, Colo205, n=4 other cells) (**m**) Cell cycle analysis by PI FACS for cell lines treated for 5d with their respective inhibitors. (cell cycle distributions determined from n=3 independent biological replicates for A375 and BT474 and n=4 for other cells; **** $p<2x10^{-16}$). (**n**) GSEA across time in longitudinal RNA-seq of $EGFR^{mut}$ cells treated with osimertinib (300 nM). (y-axis: normalized enrichment score (NES), x-axis=treatment time). Significance calculated by ANOVA with Tukey post hoc test (c,i), *t* tests (l), Cochrane-Mantel-Haenszel test (m). All tests were performed two-sided. Source data are provided as a Source Data file.

Supplementary Fig. 2

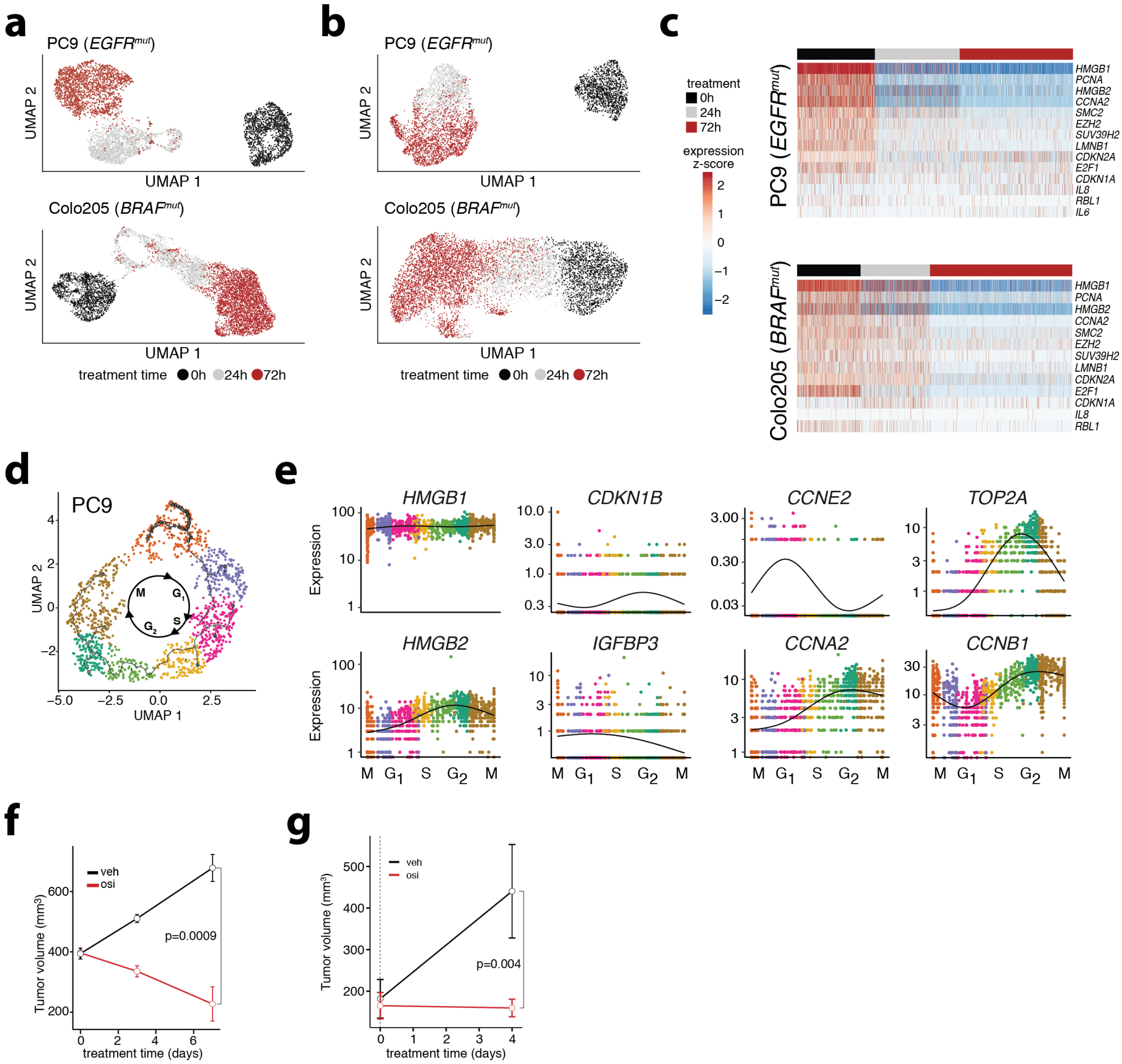

Supplementary Fig. 2: Induction of transcriptional programs by kinase inhibition.

(**a, b**) UMAP-clustering of scRNA-seq at different treatment time-points of EGFR^{mut} PC9 (osi 300nM, top) and BRAF^{mut} Colo205 (1 µM vemurafenib, bottom) based on the most variable genes (a) or based on genes of the ATSC gene set (B). (**c**) scRNA-seq expression per gene (rows, expression is scaled per row) in individual osimertinib-treated PC9 (top) or vemurafenib-treated Colo205 (bottom) cells (columns) with respect to treatment duration for senescence-repressed genes. (**d**) Clustering of cells based on scRNA-seq expression of G1/S and G2/M genes [32] recapitulates cell cycle distribution in untreated PC9 cells. (**e**) Expression across cell cycle phases confirms expected known cell cycle dependent genes CCNE2, TOP2A, CCNA2 and CCNB1, but is not present for senescence-/SASP-associated genes IGFBP3, HMGB1, HMGB2 or CDKN1B. (**f**) Tumor volume of EGFR^{mut} PDX models treated with osimertinib for 0d, 1d, 10d or vehicle for 10d (total n=8 mice, one tumor per mouse, Mean+SEM). (**g**) Tumor volumes of humanized PC9 xenografts shown in Fig. 1f-i treated for 4d with

osimertinib (5mg/kg p.o./d, n=8 tumors) or vehicle (n=7 tumors) (Mean+SEM of tumor volumes). Significance calculated by two-sided *t* tests (f, g). Source data are provided as a Source Data file.

Supplementary Fig. 3

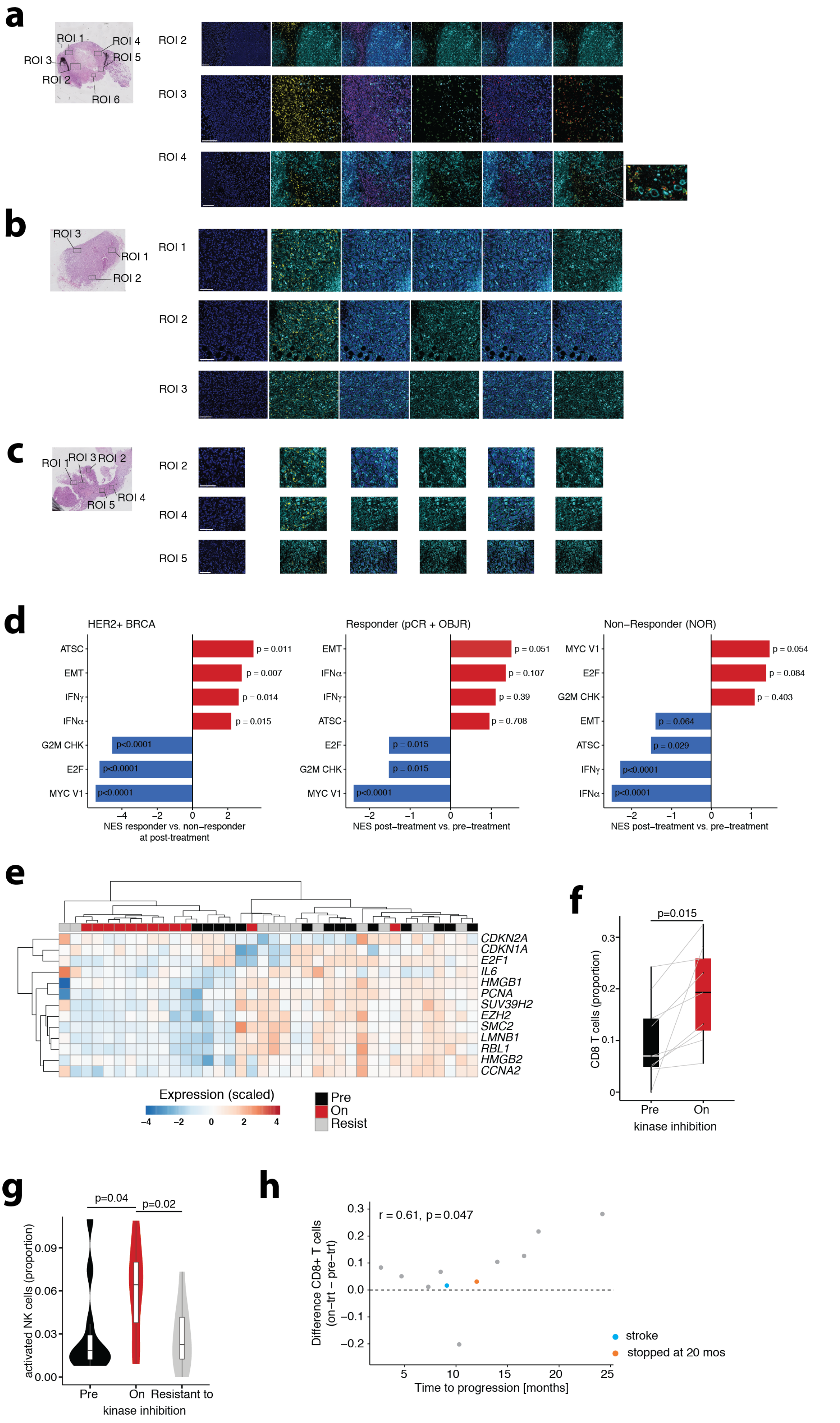

Supplementary Fig. 3: Effects on the tumor microenvironment by kinase inhibition

(**a**-**c**) Hyperion Imaging Mass Cytometry false-color images for three samples of humanized PC9 xenografts treated with osimertinib 4d (a), vehicle 4d (b) or a d0 control (c). HE and analyzed Regions Of Interest (ROI)s shown on the right, for representative ROIs staining of DNA (blue), pan-Cytokeratin (cyan), Ki67 (yellow), CD3 (magenta), CD8 (green) Granzyme B (red) are shown. Overlay of CD8 and Granzyme B yields yellow staining in the rightmost column. Scale bar 100µM, scanning was performed on multiple ROIs per tumor with one tumor per time-point & treatment group (**d**) GSEA of public gene expression data for HER2-positive breast cancer (BRCA) patients that did or did not responded to neo-adjuvant anti-HER2 therapy with the monoclonal antibody trastuzumab. Shown are the post-treatment differences between responders and non-responders (left), and pre-post therapy analyses for patients with objective response (OBJR) or pathological complete response (pCR) (middle) or non-responders (NOR, right). Significance calculated as FDR-corrected q-values (**e**) Heatmap of senescence-repressed genes [30] in RNA-seq data of $BRAF^{mut}$ melanoma patients [34] before (Pre, n=14) or during (On, n=12) BRAFi or BRAFi+MEKi treatment or after development of resistance (Resist, n=12). (**f**) Proportion of CD8 T cells inferred from bulk RNA-seq in matched pre- and on-treatment biopsies from $\mathrm{BRAF}^{\mathrm{mut}}$ melanoma patients (n=9). (**g**) Inferred proportion of activated NK cells in unmatched melanoma biopsies from patients pre-therapy (n=11), on therapy (n=11) or resistant to therapy (n=10). (**h**) Association between time to progression vs. increase of CD8 cells during therapy in melanoma patients with matched samples. Significance calculated by two-sided paired (f) or two-sided unpaired *t* test (g) and Pearson correlation (h). Boxplots display median (center line), 25th/75th percentile (lower/upper box hinges), whiskers extend to the most extreme value within 1.5× interquartile range (IQR) of the hinges. Source data are provided as a Source Data file.

Supplementary Fig. 4

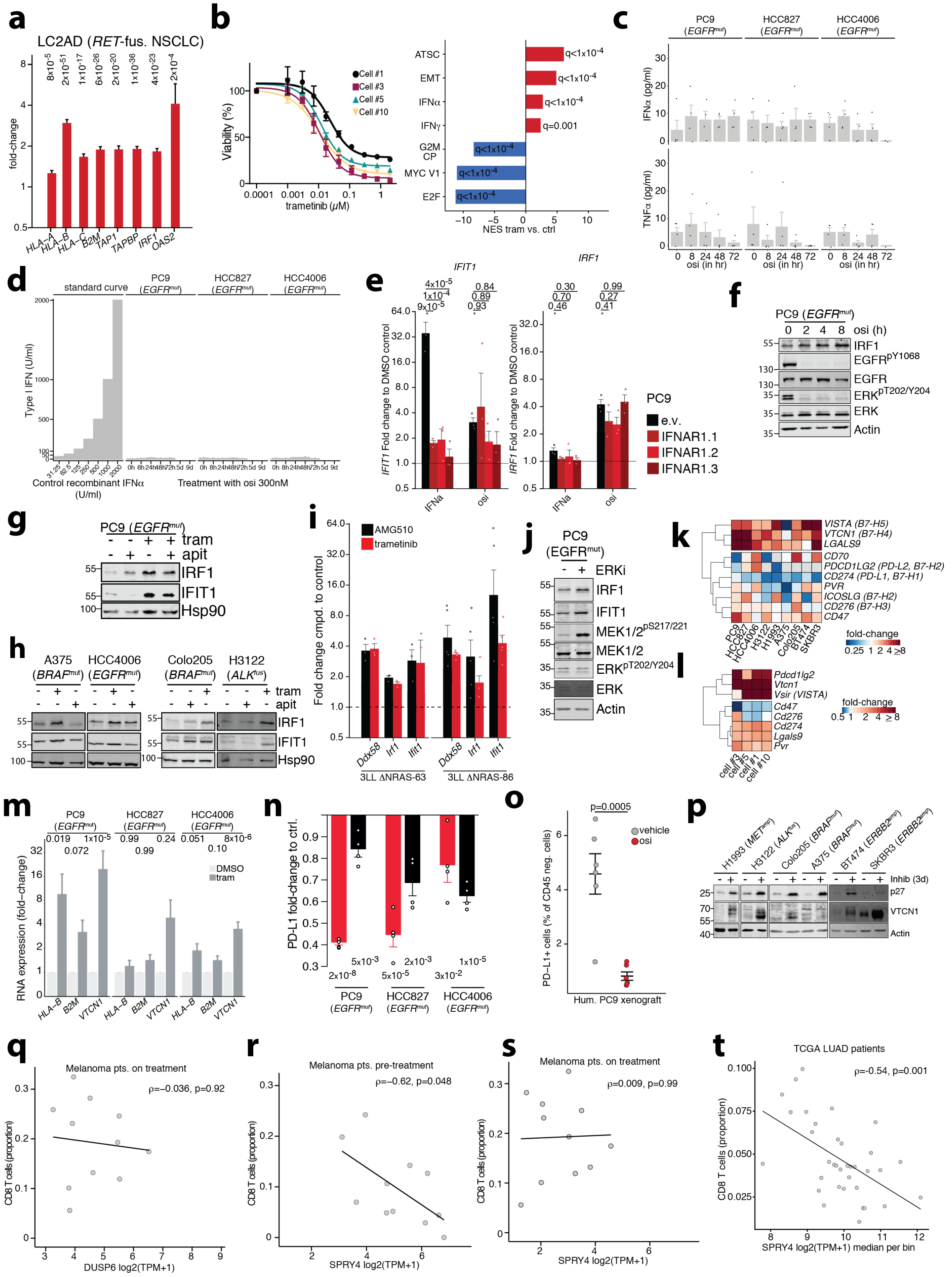

Supplementary Fig. 4: MAPK-pathway mediates inflammatory signaling and immune escape.

(**a**) Differential gene expression (DE) focusing on inflammatory genes between Ret-inhibitor AD80 treated and DMSO controls cells of the *RET^{fus}* NSCLC cell line LC2AD. (x-axis: fold-change treated vs. control, error bar = standard error of the fold-change from DE analysis, bar height indicates average fold-change; DE performed on data of n=2 treated and n=2 untreated samples with Benjamini-Hochberg adjusted p-values from two-sided DE analysis) (**b**) Left: Viability assay of primary *KRAS^{mut}* PDAC GEMM cell lines treated with trametinib for 72h as % of DMSO controls (Mean+SEM of n=3 (cells #3, #10) or n=2 (cells #1, #5) biological replicates, each performed in triplicate). Right: GSEA based on RNA-seq of PDAC cell lines treated at their respective GI_{50} for 48h. FDR-corrected q-values of Kolmogorov Smirnov based permutation test are indicated. (**c**) ELISA for IFNα and TNFα in cell culture supernatant of osimertinib-treated EGFR^{mut} cells. (Mean+SEM of n=4 biological replicates each except for HCC4006 72h only n=2) (**d**) Detection of type I IFN in cell culture supernatant of osimertinib-treated EGFR^{mut} cells using THP1-Dual reporter cell line. (Mean+SEM of n=4 independent biological replicates) (**e**) qPCR-Analysis of IFIT1 (left) and IRF1 (right) in PC9 single-cell clones after treatment IFNAR1 knock-out and PC9 e.v. controls. Fold change after treatment with osimertinib or IFNα for 24h compared to controls is shown Expression was normalized to GAPDH and fold-change calculated to controls as 2^ddCt. (Mean+SEM of n=3 independent biological replicates). (**f**) Immunoblot for time-series of protein dynamics in PC9 cells during osimertinib treatment. Representative image of n=3 independent experiments. (**g,h**) Protein levels of interferon regulatory factor 1 (IRF1) and interferon inducible target 1 (IFIT1) in cells treated with MEK (trametinib, 100 nM) or PI3K (apitolisib, 500nM) inhibitors. Representative images of n=3 independent experiments. (**i**) RT-qPCR analysis of gene expression in murine KRAS^{mut} Lewis Lung carcinoma cell line (3LL) with NRAS knock-out [40] treated with KRAS-inhibitor AMG510 (1 µM) or trametinib (100 nM) for 72 h. Expression was normalized to β-Actin and fold-change calculated to controls as 2^ddCt. (Mean+SEM of independent biological replicates with n=3 3LL ΔNRAS-63, n=5 for 3LL ΔNRAS-86) (**j**) Immunoblot of PC9 cells treated with ERK-inhibitor SCH772984 (500nM, 72h). Representative image of n=3 independent experiments. (**k,l**) RNA-seq based expression of inhibitory immune checkpoints in human cell lines treated with their respective kinase inhibitors for 72 h (k) or primary *KRAS^{mut}* PDAC cell lines treated with trametinib for 48 h (l). Colors indicate fold-changes to untreated controls. (**m**) RNA-seq expression of antigene-presenting machinery genes in EGFR^{mut} cells following MEK-inhibition with trametinib (100 nM, 3d). (x-axis: fold-change treated vs. control, error bar = standard error of the fold-change from DE analysis, bar height indicates fold chance from n=2 untreated and n=2 treated samples per cell line; p-values from differential expression test adjusted for multiple testing using Benjamini-Hochberg correction) (**n**) Flow-cytometry analysis of PD-L1 surface expression of EGFRmut cell lines treated with osimertinib (300nM, 72h) or DMSO controls. (Mean+SEM,n=4 biological replicates). (**o**) Flow-cytometry anylsis of PD-L1 positive, CD45-negative cells in humanized PC9 xenografts after 4d treatment. (each data point = one tumor, max. 2 tumors per mouse, error bars represent mean±SEM of n=6 tumors per group). (**p**) Expression of p27 and VTCN1 across oncogene-driven tumor cell lines treated with their respective kinase inhibitor. (**q-s**) Correlation of RNAseq-based CD8 T cell proportion with expression of negative MAPK feedback genes *DUSP6* and *SPRY4* in untreated *BRAF^{mut}* melanoma patients (n=11, pre-treatment) or during BRAF- and/or MEK-inhibition (n=11, on treatment). Representative image of n=3 independent experiments. (**t**) Correlation of *SPRY4* expression in TCGA lung adenocarcinoma patients with valid CIBERSORT cell type estimates (n=350). Patients were binned by *SPRY4* expression, median log2(TPM+1)

and median CD8 T cell proportion per bin are displayed. Significance calculated ANOVA and Tukey post-hoc test (e), *t* tests (n, o) and correlation calculated with Spearman rank correlation (q-t). All tests performed two-sided. Source data are provided as a Source Data file.

Supplementary Fig. 5

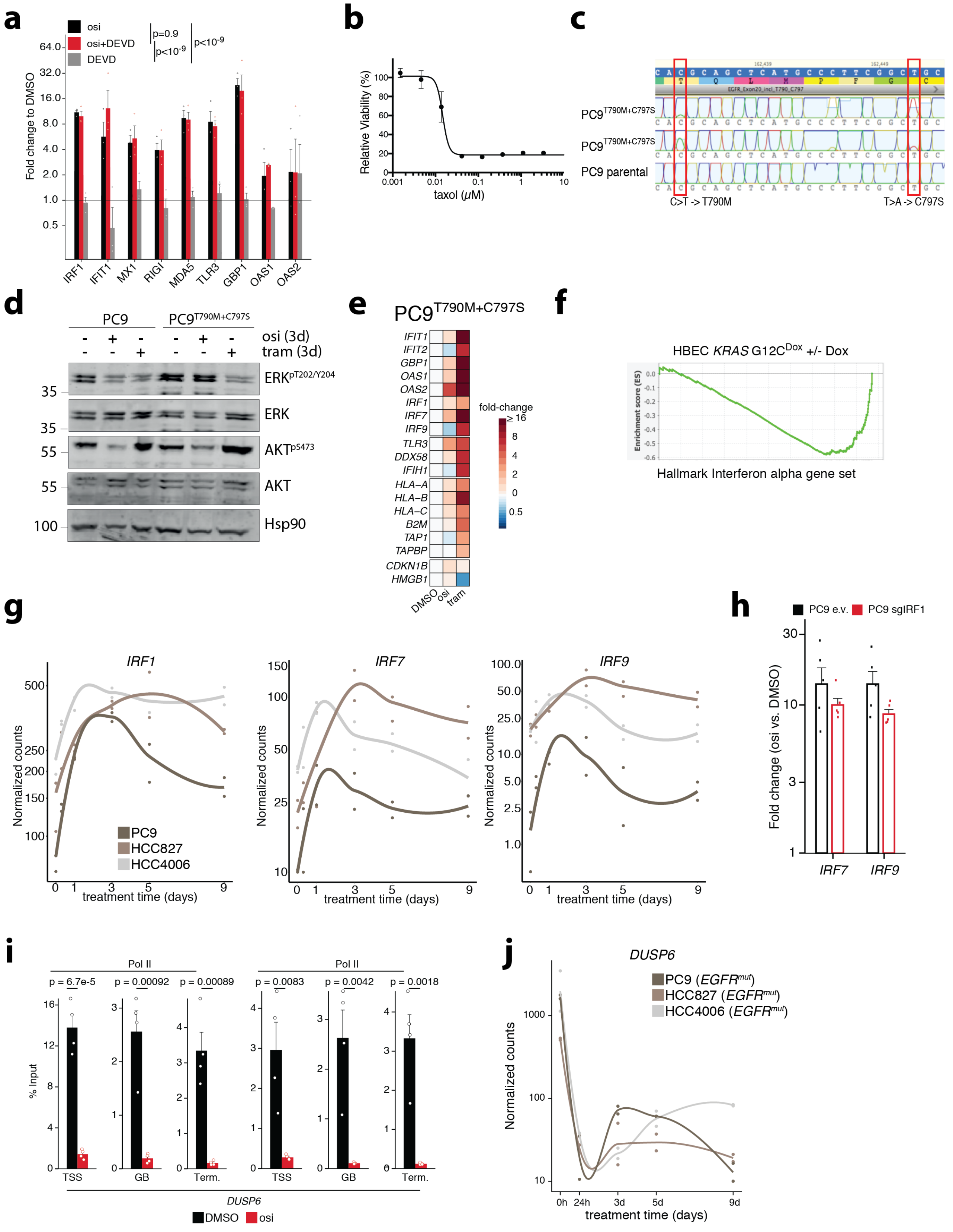

Supplementary Fig. 5: Inflammatory transcription is driven by MAPK-IRF1 axis.

(**a**) RT-qPCR analysis of IFN-target genes of PC9 cells treated with osi (300 nM), Caspase3/7 inhibitor (DEVD; Z-DEVD-FMK, 50 μM) or a combination of both. Fold-change compared to DMSO controls. (Mean+SEM of n=3 independent biological replicates) (**b**) CTG assay of $EGFR^{mut}$ PC9 cells treated with taxol for 72h compared to DMSO control (Mean±SEM of n=3 biological replicates, each in triplicate). (**c**) Sanger sequencing of the EGFR-allele in PC9 parental and the CRISPR-engineered $PC9^{T790M+C797S}$ to validate the introduced mutations. (**d**) Immunoblot of PC9 parental and $PC9^{T790M+C797S}$ following trametinib or osimertinib to assess sustained AKT and MAPK signaling in $PC9^{T790M+C797S}$. Representative image of n=3 independent biological replicates. (**e**) RNA-seq based gene expression of inflammatory genes in $PC9^{T790M+C797S}$ following treatment with osimertinib (300 nM, 3 d) or trametinib (100 nM, 3 d) as fold-change to DMSO controls. (**f**) Enrichment plot of the MSigDB Hallmark Interferon alpha gene set in public data[47] human bronchial epithelial cells (HBEC) carrying a Doxycycline-inducible *KRAS* G12C mutation after Doxycycline treatment vs. controls. (**g**) Time-series RNA-seq based expression of Interferon-regulating factors (IRFs) 1, 7 and 9 in three $EGFR^{mut}$ cell lines during osimertinib treatment (300 nM). (**h**) RT-qPCR analysis of IRF7 and IRF9 in PC9 e.v. or IRF1 knock-out PC9 cells after 72h osimertinib treatment. Fold-change to DMSO control. (Mean+SEM of n=5 biological replicates) (**i**) ChIP-qPCR analysis of total Pol II or phosphorylated Pol II pSer2/5 RNA Pol II binding to the transcription start site (TSS), gene body (GB) and transcription termination site (Term.) of *DUSP6* compared to input control after 72 h osimertinib (300 nM) or DMSO treatment (Mean+SEM of n=4 independent biological replicates). (**j**) Time-series RNA-seq based expression of DUSP6 in three $EGFR^{mut}$ cell lines during osimertinib treatment (300 nM). Significance was calculated using two-way ANOVA adjusting for gene differences followed-by Tukey post hoc test (a) and t tests (i) not adjusted for multiple comparisons. All tests performed two-sided. Source data are provided as a Source Data file.

Supplemenatry Fig. 6

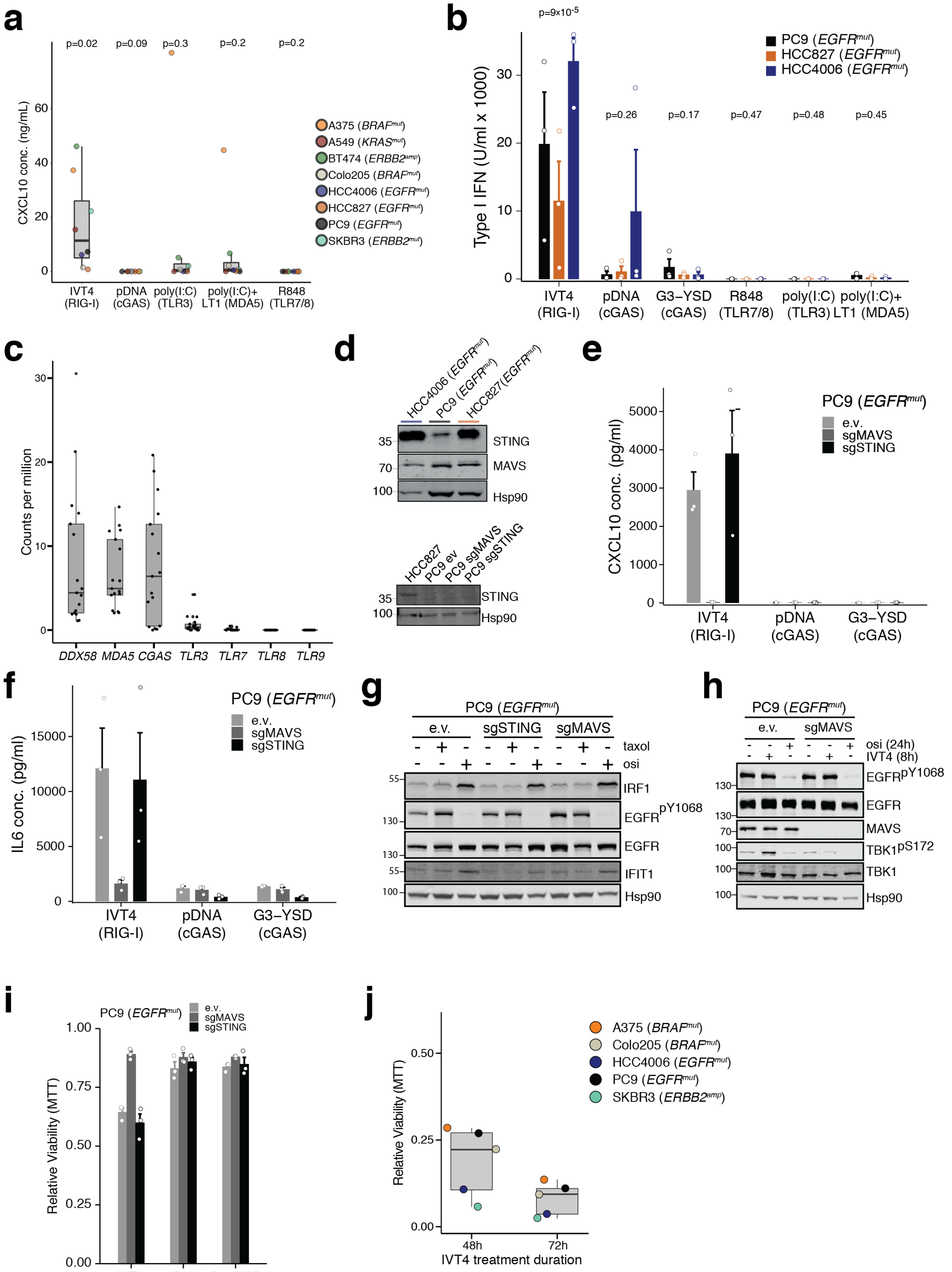

Supplementary Fig. 6: Activation of nucleic acid sensing machinery induces secretion of inflammatory cytokines and impairs cell growth in kinase-driven cancer cells.

(**a**) ELISA of secreted CXCL10 in supernatant of oncogene-driven cancer cell lines following 16 h stimulation with nucleic acid receptor agonists. (Mean+SEM of n=3 independent biological replicates) (**b**) Measurement of type I interferon secretion using the THP1-Dual reporter cell line after stimulation as in (a). (Mean+SEM of n=3 independent biological replicates) (**c**) RNA-seq based expression of nucleic acid receptors stimulated with agonists in cell lines in (a) and (b) (n=17 RNA-seq samples of n=8 cell lines). (**d**) Steady-state expression of MAVS and STING in $EGFR^{mut}$ cell lines. Representative images of n=3 independent biological replicates. (**e**, **f**) Inducible secretion of IL6/CXCL10 in PC9 cells carrying a lentiCRISPRv2 e.v. or an sgRNA targeting MAVS or STING. (Mean+SEM of n=3 independent biological replicates) (**g**) Immunoblot of PC9 cells carrying CRISPRv2 e.v. or sgRNAs targeting MAVS or STING as DMSO controls and after treatment with taxol (30 nM) or osimertinib (300 nM) for 48 h. Representative images of n=3 independent biological replicates. (**h**) Immunoblot of MAVS knock-out and e.v. control PC9 after treatment with DMSO/osimertinib (300nM) and IVT4 or IVT-GAC (1 ng/µL). Representative images of n=3 independent biological replicates. (**i**) MTT assay for relative viability in PC9 with *MAVS* or *STING* knock-out. (Mean+SEM of n=3 independent biological replicates). (**j**) Relative viability of kinase-driven cells lines treated with IVT4 for 48h or 72h compared to untreated controls (Mean+SEM of n=3 independent biological replicates). Significance was calculated by one-sample t tests (a) and agonist vs. control adjusted for cell line by two-way ANOVA (b). Boxplots display median (center line), 25th/75th percentile (lower/upper box hinges), whiskers extend to the most extreme value within 1.5× interquartile range (IQR) of the hinges. Source data are provided as a Source Data file.

Supplementary Fig. 7

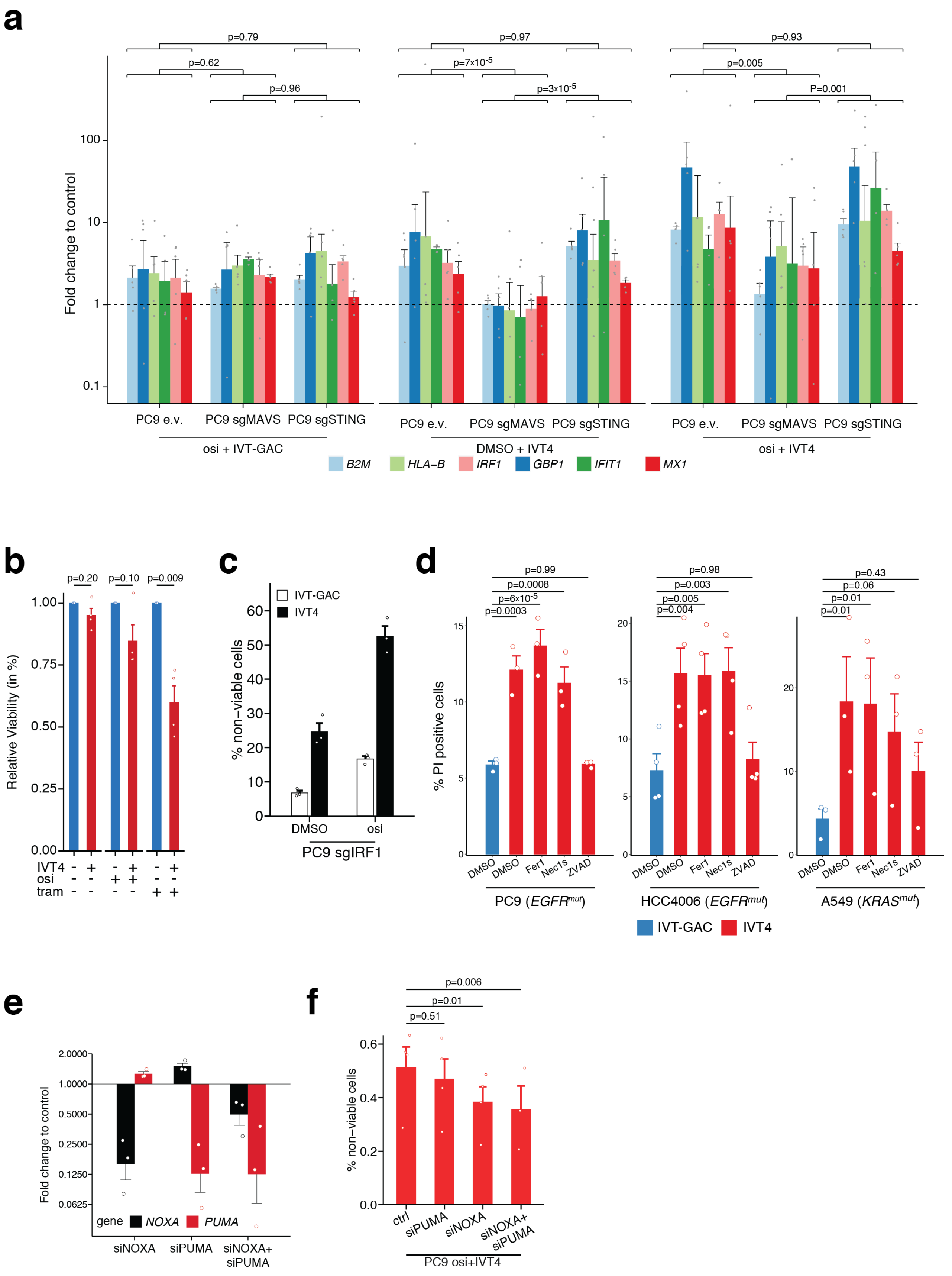

Supplementary Fig. 7: Targeted kinase-inhibition enhances NAR agonist induced cell death.

(**a**) RT-qPCR analysis of inflammatory genes in e.v., sgMAVS or sgSTING PC9 cells treated with DMSO or osimertinib (300nM) and IVT4 (1ng/µL) or IVT-GAC (1ng/µL) or combinations for 24h. Fold-changes calculated to DMSO+IVT-GAC controls. (Mean+SEM of n=5 independent biological replicates) (**b**) CTG assay for PC9$^{T790M+C797S}$ pre-treated for 48h with trametinib (100 nM) or osimertinib (300 nM) and subsequent addition of IVT4/IVT-GAC for 24h. (Mean+SEM of n=4 independent biological replicates). (**c**) Flow-cytometric analysis of cell death induction following 24 h treatment of PC9 cells carrying CRISPRv2 e.v. or sgIRF1 with DMSO/osimertinib (300nM) and IVT4/IVT-GAC (vertical axis displays normalized percentage of AnnexinV and/or PI positive cells = % cell death) (Mean+SEM of n=3 independent biological replicates). (**d**) Flow-cytometry with PI staining to analyze cell death in cells treated for 24h with IVT-GAC or IVT4 (1 ng/µL) in combination with cell death inhibitors Fer1 (5µM), Nec1s (10µM) or ZVAD-FMK (20µM). (Mean+SEM of independent biological replicates, n=3 for PC9 and A549, n=4 for HCC4006). (**e**) RT-qPCR analysis of NOXA and PUMA in PC9 cells after 72h treatment with siRNA against *NOXA*, *PUMA* or both. Fold-change compared to controls. (Mean+SEM of n=3 independent biological replicates). (**f**) Flow-cytometry with PI staining to analyze cell death in PC9 cells pre-treated with indicated siRNA for 48h followed by 24h treatment of osimertinib (300nM) with IVT4 (1 ng/µL). (Mean+SEM of independent biological replicates, n=3 for siNOXA+siPUMA, n=4 otherwise). Significance was calculated by two-way ANOVA with Tukey post hoc tests adjusting for baseline genes on log fold changes (a), ANOVA with Tukey post hoc test (f), two-way ANOVA adjusting for batch followed by Tukey post hoc tests (d) and t tests (b). All tests performed two-sided. Source data are provided as a Source Data file.

Supplementary Fig. 8

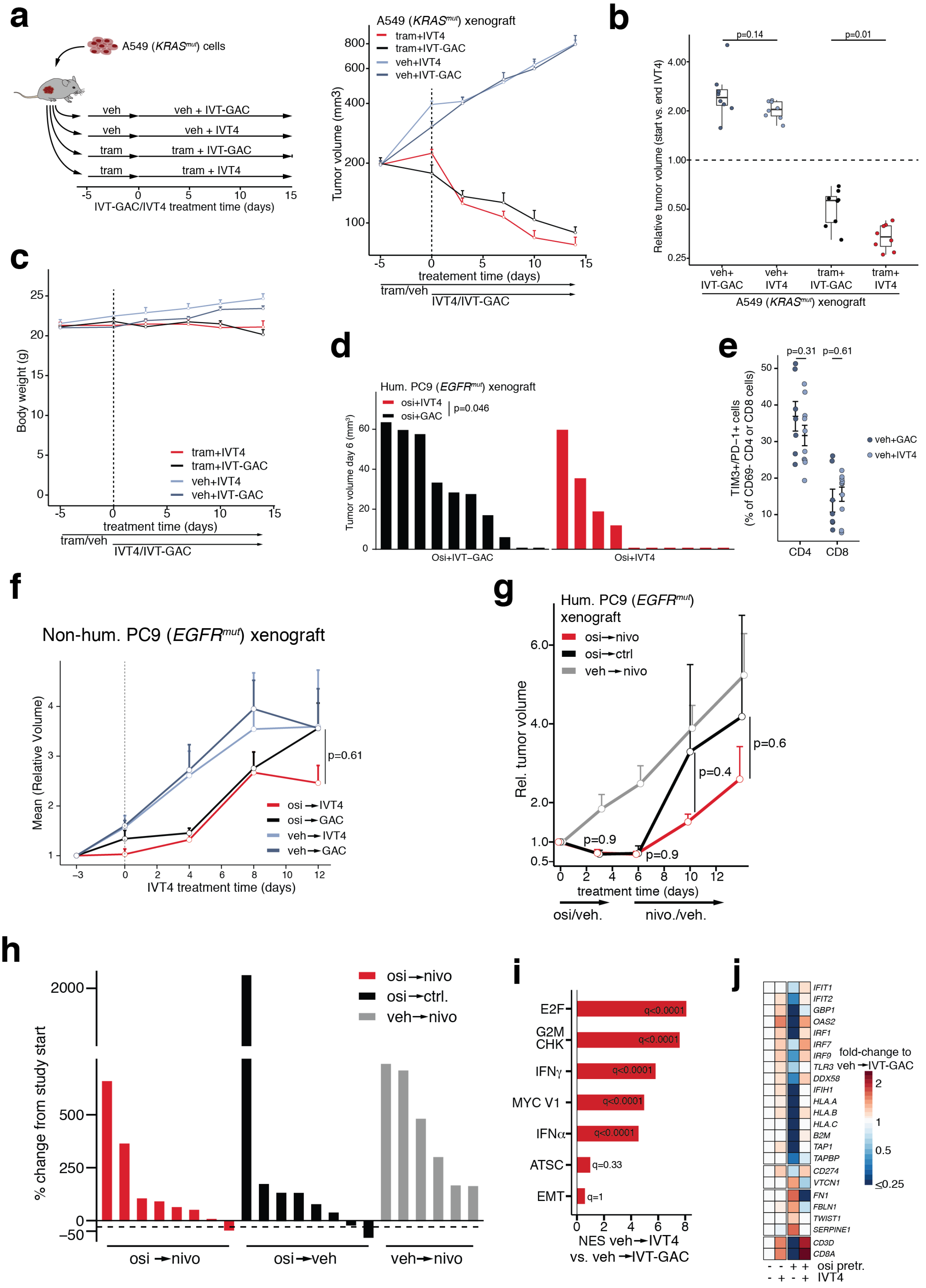

Supplementary Fig. 8: RIG-I agonist enhances efficacy of targeted treatment in vivo.

(**a**) Schematic overview (left) and tumor volumes (right) of A549 xenografts treated with daily trametinib (3mg/kg p.o.) or vehicle with addition of IVT4/IVT-GAC i.t. starting on day 5 (Mean+SEM; n=8 tumors per group, 2 tumors per mouse). (**b**) Relative tumor volumes of A549 xenografts from (a) after 14 d combined treatment with vehicle/trametinib (3 mg/kg p.o.) and IVT4/IVT-GAC i.t. compared to start of IVT4/IVT-GAC treatment. (n=8 tumors of n=4 mice) (**c**) Body weight of mice from (a,b) (Mean+SEM; n=4 mice per group). Boxplots display median (center line), 25th/75th percentile (lower/upper box hinges), whiskers extend to the most extreme value within 1.5× interquartile range (IQR) of the hinges. (**d**) Tumor volumes of a cohort of humanized PC9 xenografts at the time of harvest after 4d osimertinib and 6 days of IVT4 or IVT-GAC treatment. (each bar is one tumor, max. 2 tumors per mouse) (**e**) Flow-cytometry of tumor-infiltrating CD4 and CD8 lymphocytes for TIM3 and PD1 expression after 4d vehicle and 6d IVT4/IVT-GAC. (each data point = one tumor, max. 2 tumors per mouse, error bars represent mean±SEM of n=7 vehi+GAC or n=10 veh+IVT4 tumors). (**f**) Relative tumor volumes in a non-humanized PC9 xenograft treated with osimertinib/vehicle followed by IVT4 or IVT-GAC (Mean+SEM, n=20 mice, 1-2 tumors per mouse). (**g**) Humanized PC9 xenografts were pre-treated with osimertinib (5 mg/kg p.o.) before being switched to anti-PDL1 antibody treatment i.p. Tumor volumes are normalized to treatment start. (Mean+SEM of n=8 osi+vehicle, n=8 osi+nivolumab or n=6 vehicle+nivolumab treated tumors) (**h**) Percent change of tumor volumes between start and end of the osimertinib-ICB study in humanized PC9 xenografts. (**i**) RNA-seq based GSEA of humanized mice treated with vehicle followed by IVT4 or vehicle followed by IVT-GAC. (Significance calculated as FDR-adjusted q-values by Kolmogorov Smirnov based permutation test) (**j**) RNA-seq fold-changes in humanized mice per treatment arm compared to vehicle-IVT-GAC control animals. Significance calculated by ANOVA adjusting per mouse in treatment arms (b,d,g) and t tests (e). All tests two-sided. Source data are provided as a Source Data file.

Supplementary Fig. 9

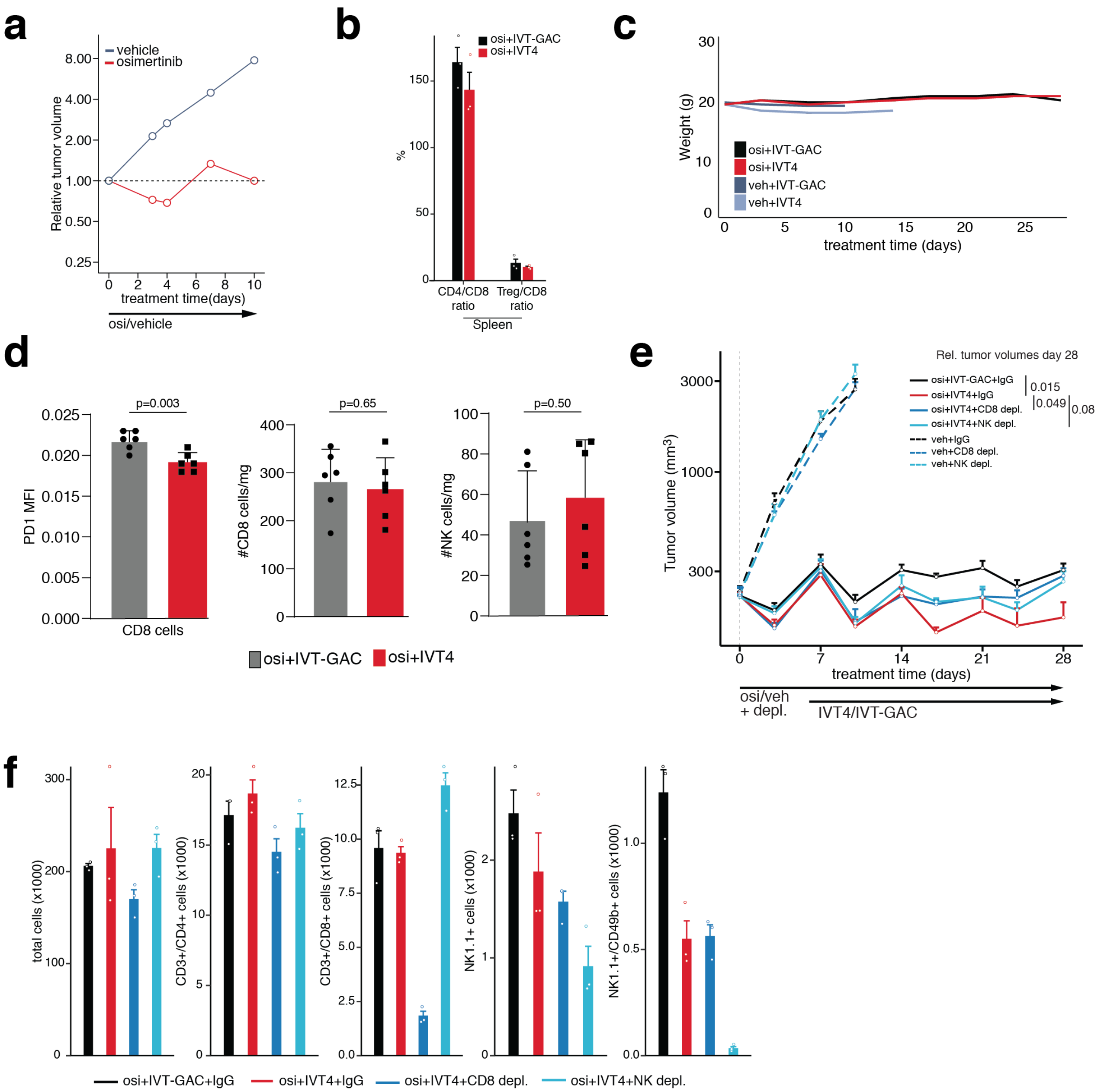

Supplementary Fig. 9: Combined kinase inhibitor and RIG-I agonist treatment in a syngeneic Egfr^{mut} mouse model.

(**a**) Relative tumor volume of syngeneic *Egfr^{mut}* mouse model treated for 0d, 4d or 10d with osi or 10d with vehicle (Mean, each time point n=2 mice, both tumors per mouse were averaged). (**b**) Ratio of splenic CD4, T_{reg} and CD8 T cells in syngeneic *Egfr^{mut}* mice treated with osi and IVT4 or IVT-GAC control. (Mean+SEM, n=3 three mice per group) (**c**) Body weight of syngeneic *Egfr^{mut}* mice treated with osimertinib. (**d**) Flow-cytometry analysis of number of tumor infiltrating CD8 or NK cells per mg tissue processed after 6d osimertinib followed by 10d osimertinib + IVT4 or IVT-GAC. (Mean+SD; each data point = one tumor, 2 tumors per mouse; MFI = mean fluorescence intensity). (**e**) Tumor growth of syngeneic *Egfr^{mut}* mice under osimertinib/vehicle treatment combined with IVT4/IVT-GAC and depletion of CD8 cells, NK cells or IgG2 controls. (Mean+SEM of n=6 tumors in n=3 mice per group) (**f**) Analysis of splenic cell type composition of syngeneic *Egfr^{mut}* after treatment with

osimertinib and IVT4 including cell type depletion arms. (Mean+SEM, n=3 mice per treatment group). Significance calculated by two-samples (a, d) or one-sample (f) *t* tests or ANOVA (e). Source data are provided as a Source Data file.

Supplementary Fig. 10

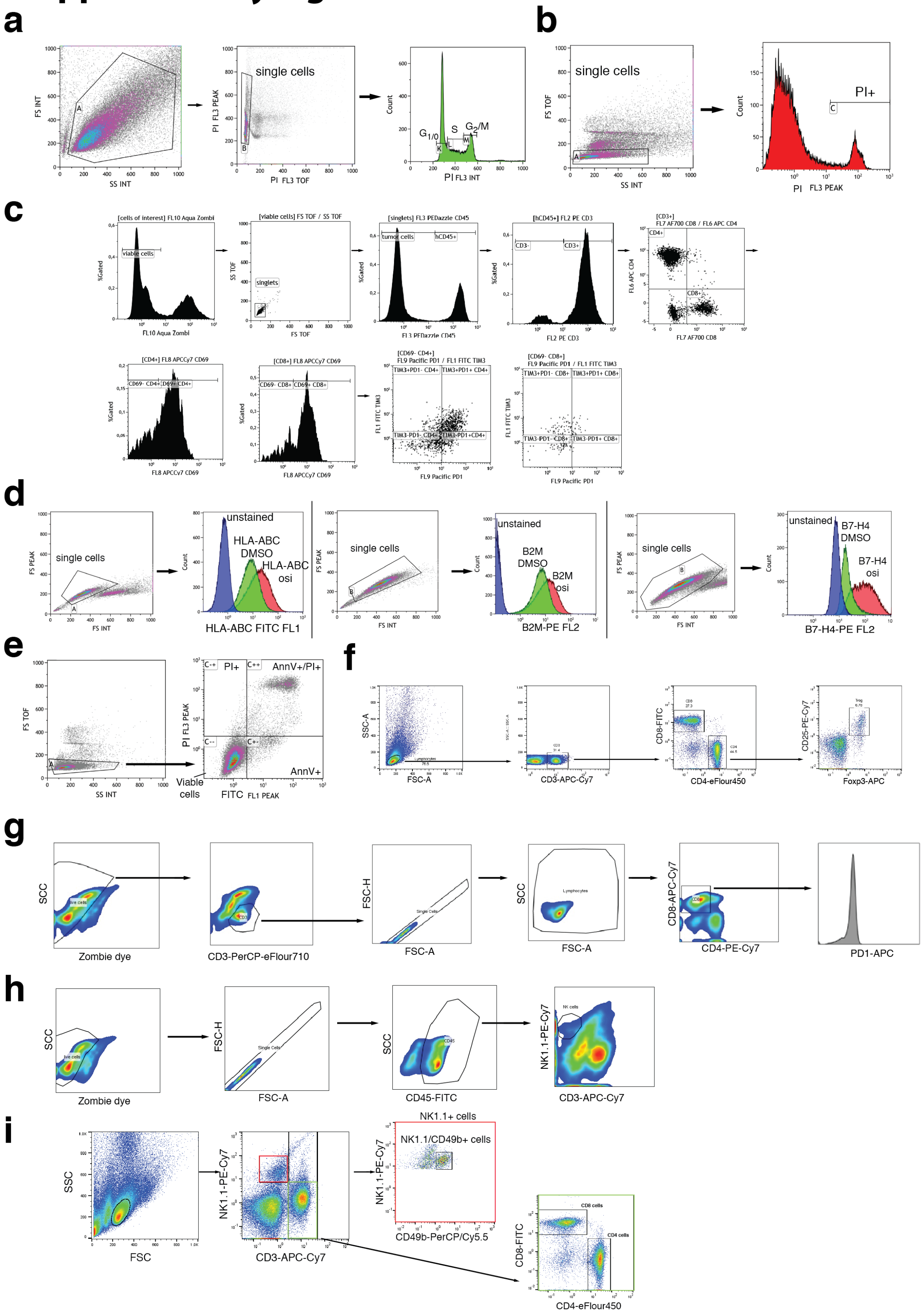

Supplementary Fig. 10: Gating strategies used in flow-cytometry experiments

(**a**) For cell cycle analyses debris and cell aggregates were removed followed by gating on the PI signal to determine cell cycle phases in Fig. 1b and Supplementary Figs. 1e,m. (**b**) To determine dead cells by PI incorporation single cells were gated to exclude debris and cell aggregates followed by gating on PI+ vs. PI- cells in Supplementary Figs. 1d, 7d,f. (**c**) Sequential gating to determine the fraction of exhausted T cells in tumors extracted from humanized mice in Figs. 1i, 5f and Supplementary Fig. 8e. (**d**) To determine surface expression of proteins HLA, B2M, VTCN1 and PD-L1 first cell aggregates and debris were removed followed by measurement of the respective fluorescent intensities per sample (overlay histogram of three samples shown) (Fig. 2e and Supplementary Fig. 4n) (**e**) Gating strategy to distinguish proportions of viable and dead/apoptotic cells in Annexin V/PI assay with removal of debris and cell aggregates followed by measurement of PI and AnnV-FITC fluorescence (Figs. 5a,c,d, Supplementary Fig. 7c). (**f**) To determine lymphocyte subsets in spleens of syngeneic $Egfr^{mut}$ mice treated with osi+/-IVT4 it was gated on single cells followed by sequential gating on CD3+, then CD8+ or CD4+ cells followed by CD25/Foxp3. (Supplementary Fig. 9b) (**g**) Gating strategy to determine amount of CD8 cells and PD-1 expression on CD8 T cells in tumors extracted from syngeneic $Egfr^{mut}$ mice by excluding dead cells, gating on single CD3 cells followed by determination of CD8+PD1+ cells. (Supplementary Fig. S9d left, middle) (**h**) To determine amount of NK cells in tumors extracted from syngeneic $Egfr^{mut}$ mice dead cells and debris were excluded folled by determination of CD45+/NK1.1+/CD3- cells (Supplementary Fig. S9d, right) (**i**) To determine the amount of NK, CD4 and CD8 cells in spleens of syngeneic $Egfr^{mut}$ mice following depletion of cell populations single cells were determined and debris excluded followed by gating for CD3-/NK1.1+, CD3-/NK1.1+/CD49b+ or CD3+/CD8+ or CD3+/CD4+ cells (Supplementary Fig. 9f).
