# Supplementary material for: MAPK-pathway inhibition mediates inflammatory reprogramming and sensitizes tumors to targeted activation of innate immunity sensor RIG-I
Source: Nat Commun. 2021 Sep 17;12:5505. doi: 10.1038/s41467-021-25728-8 (PMC8448826; doi:10.1038/s41467-021-25728-8)
Supplement: Supplementary file 3 — Reporting Summary [file 41467_2021_25728_MOESM3_ESM.pdf]

nature research

Corresponding author(s): Martin Sos, Johannes Brägelmann

Last updated by author(s): Aug 15, 2021

Reporting Summary

Nature Research wishes to improve the reproducibility of the work that we publish. This form provides structure for consistency and transparency in reporting. For further information on Nature Research policies, see our Editorial Policies and the Editorial Policy Checklist.

Statistics

For all statistical analyses, confirm that the following items are present in the figure legend, table legend, main text, or Methods section.

n/a	Confirmed	
☐	☒	The exact sample size (n) for each experimental group/condition, given as a discrete number and unit of measurement
☐	☒	A statement on whether measurements were taken from distinct samples or whether the same sample was measured repeatedly
☐	☒	The statistical test(s) used AND whether they are one- or two-sided<br>*Only common tests should be described solely by name; describe more complex techniques in the Methods section.*
☒	☐	A description of all covariates tested
☐	☒	A description of any assumptions or corrections, such as tests of normality and adjustment for multiple comparisons
☐	☒	A full description of the statistical parameters including central tendency (e.g. means) or other basic estimates (e.g. regression coefficient) AND variation (e.g. standard deviation) or associated estimates of uncertainty (e.g. confidence intervals)
☐	☒	For null hypothesis testing, the test statistic (e.g. F, t, r) with confidence intervals, effect sizes, degrees of freedom and P value noted<br>*Give P values as exact values whenever suitable.*
☒	☐	For Bayesian analysis, information on the choice of priors and Markov chain Monte Carlo settings
☒	☐	For hierarchical and complex designs, identification of the appropriate level for tests and full reporting of outcomes
☐	☒	Estimates of effect sizes (e.g. Cohen's d, Pearson's r), indicating how they were calculated

Our web collection on statistics for biologists contains articles on many of the points above.

Software and code

Policy information about availability of computer code

Data collection	Data were acquired ImageStudie v.5.2.5 (LI-COR Bioscience), Tecan i-control v.3.9.1 (Tecan, Austria), Gallios software 1.2 (Beckman Coulter, Brea, CA, US), Diva software 8.0 (BD Bioscience), MACSQuantify v.2.13
Data analysis	Data were analyzed with Kaluza Analysis Software Version 2.1, FlowJo v10.2, GraphPad Prism v6.0, MCD Viewer v1.0.560 (Fluidigm, USA), GSEA v4.0.3 (MIT Broad Institute, USA), Integrated Genome Viewer v2.3.68, RSEM v1.3.1, STAR v2.7.0, samtools v1.3, CIBERSORT, Bowtie2 v2.2.5, Cellranger Pipeline v2.0.1 (10x Genomics, USA) salmon v0.12 and the statistical programming environment R v3.5.0 including packages DESeq2 v1.18, TXImport v1.6, pheatmap v1.0.10, dplyr v1.0.2, tidyr v1.1.2, GSVA v1.30.0, Seurat v3.0.2.

For manuscripts utilizing custom algorithms or software that are central to the research but not yet described in published literature, software must be made available to editors and reviewers. We strongly encourage code deposition in a community repository (e.g. GitHub). See the Nature Research guidelines for submitting code & software for further information.

Data

Policy information about availability of data

All manuscripts must include a data availability statement. This statement should provide the following information, where applicable:

- Accession codes, unique identifiers, or web links for publicly available datasets
- A list of figures that have associated raw data
- A description of any restrictions on data availability

The RNA-seq data of murine PDAC cells generated in this study have been deposited in the Gene Expression Omnibus database under accession code GSE181599 (www.ncbi.nlm.nih.gov/geo/). All other sequencing data has been deposited in the ArrayExpress database under accession codes E-MTAB-9468, E-MTAB-9883, E-MTAB-9884, E-MTAB-9885, E-MTAB-9887, E-MTAB-9888, E-MTAB-9889, E-MTAB9890, E-MTAB9891, E-MTAB-9886, E-MTAB-10851 and E-MTAB10802. We also used the publicly accessible data sets GSE76360 (breast cancer RNA expression), GSE100336 (KRAS G12C expression data) (www.ncbi.nlm.nih.gov/geo/) and

EGAS00001000992 (melanoma patient RNA-seq), EGAS00001002335 (LC2/AD RNA-seq) (https://ega-archive.org). The remaining data are available within the Article, Supplementary Information or Source Data file.

Field-specific reporting

Please select the one below that is the best fit for your research. If you are not sure, read the appropriate sections before making your selection.

- [x] Life sciences
- [ ] Behavioural & social sciences
- [ ] Ecological, evolutionary & environmental sciences

For a reference copy of the document with all sections, see nature.com/documents/nr-reporting-summary-flat.pdf

Life sciences study design

All studies must disclose on these points even when the disclosure is negative.

Sample size	Sample sizes were not determined a priori. We used generally accepted sample sizes in accordance to own previous experiences with reproducable differences between conditions indicating that the chosen sample sizes were sufficient.
Data exclusions	No data were excluded.
Replication	Cellular experiments were performed in at least three independent biological replicates including technical replicates per experiment if feasible (e.g. qPCR, MTT, CTG assays). Technical replicates were averaged within each biological replicate prior to further analysis. All figures thus show averages and variability calculated from at least three independent experiments. All experimental replicates were included in the final analyses and successfully validated the experimental findings within the reported degree of variability.<br>In vivo experiments were performed across different independent cohorts and mouse models to increase biological reproducibility. Per cohort at least four independent tumors were used. Treatment experiments combining osimertinib +/-IVT4 were repeated in two independent cohorts of humanized mice or the syngeneic EGFR-mutant mouse model each with comparable results per replicate.
Randomization	For in vivo experiments allocation of tumor-bearing mice to experimental groups was done randomly before the start of treatment. For in vitro experiments cells were distributed randomly to control and experimental conditions and processed in parallel.
Blinding	For in vivo work treatment and volume measurements were distributed to different people to ensure blinding wherever possible. Moreover, researchers performing flow-cytometry of mouse xenografts were blinded to treatment group of the mice. Histology scoring was done in a blinded manner i.e. person scoring was blinded to treatments the respective animals received. For in vitro cell line work, data collection/ analysis was not blinded, but repeated by independent persons thereby reducing bias.

Reporting for specific materials, systems and methods

We require information from authors about some types of materials, experimental systems and methods used in many studies. Here, indicate whether each material, system or method listed is relevant to your study. If you are not sure if a list item applies to your research, read the appropriate section before selecting a response.

Materials & experimental systems

n/a	Involved in the study
[ ]	[x] Antibodies
[ ]	[x] Eukaryotic cell lines
[x]	[ ] Palaeontology and archaeology
[ ]	[x] Animals and other organisms
[ ]	[x] Human research participants
[x]	[ ] Clinical data
[x]	[ ] Dual use research of concern

Methods

n/a	Involved in the study
[x]	[ ] ChIP-seq
[ ]	[x] Flow cytometry
[x]	[ ] MRI-based neuroimaging

Antibodies

Antibodies used	Western blot: Actin (#sc-47778, Santa Cruz Biotechnology, 1:5000), pan-AKT (#2920, Cell Signaling, 1:1000), pAKTS473 (#9271, Cell Signaling, 1:1000), BCL2 (#2876, Cell Signaling, 1:1000), BRAFV600E (#E19290, Spring Bioscience, 1:1000), Cas9 (#14697, Cell Signaling, 1:1000), cleaved Casp.3 (#9664, Cell Signaling, 1:1000), cPARP (#55-2597, BD Bioscience, 1:1000), EGFR (#2239, Cell Signaling, 1:1000), pEGFRY1068 (#3777, Cell Signaling, 1:1000), ERK (#4696, Cell Signaling, 1:1000), pERKT202/Y204 (#4370, Cell Signaling, 1:1000), H3K27me3 (#9733, Cell Signaling, 1:1000), H3K9me3 (#C15410056, Diagenode, 1:1000), Hsp90 (#4877, Cell Signaling, 1:5000), IFIT1 (#14769, Cell Signaling, 1:1000), IRF1 (#8478, Cell Signaling, 1:1000), IRF3 (#4302, Cell Signaling, 1:1000), pIRF3S396 (#4947, Cell Signaling, 1:1000), MAVS (#3993, Cell Signaling, 1:1000), MCL1 (#5453, Cell Signaling, 1:1000), p16 (#ab108349, Abcam, 1:1000), p21 (#2947, Cell Signaling, 1:1000), p27 (#3686, Cell Signaling, 1:1000), p65 (#8242, Cell Signaling, 1:1000), pp65S5367 (#3033, Cell Signaling, 1:1000), RIG-I (#3743, Cell Signaling, 1:1000), STING (#13647, Cell Signaling, 1:1000), TBK1 (#3504, Cell Signaling, 1:1000), pTBK1S172(#5483, Cell Signaling, 1:1000), total H3 (#C15310135, Diagenode, 1:1000), VTCN1 (#14572, Cell Signaling, 1:1000); goat anti-rabbit 800CW (#926-32211, LI-COR Biosciences, 1:10000), goat anti-mouse 800CW (#926-32210, LI-COR Biosciences, 1:10000), anti-rat 680 (#925-68076, LI-COR Biosciences, 1:10000), donkey anti-rabbit 680LT (#926-68023,

LI-COR Biosciences, 1:10000), and donkey anti-mouse 680LT (#926-68022, LI-COR Biosciences, 1:10000).

Flow cytometry: B7-H4/VTCN1 (#358104, BioLegend, 1:40), beta-2 microglobulin (B2M) (#A15770, ThermoFisher Scientific, 1:100), HLA-ABC (#11-9983-42, ThermoFisher Scientific, 1:40); CD3 (17A2, #46-0032-82; #100222, 1:50), CD8 (53-6.7, #100714, 1:50), PD-1 (29F.1A12, #135210, 1:50), CD45 (30-F11, #103108, 1:50), NK1.1 (PK136, #25-5941-82, 1:50), CD3 (PE, Biolegend, UCHT1, 1:100), CD4 (APC, Biolegend, RPA-T4, 1:100), CD45 (PE/DazzleTM594, Biolegend, HI30, 1:100), CD69 (APC/Cy7, Biolegend,FN50, 1:100), CD8 (Alexa Fluor®700, Biolegend, SK1, 1:100), PD-1 (Pacific Blue, Biolegend, EH12.2H7, 1:100), PD-L1 (PE/Cy7, Biolegend, 29E.2.A3, 1:100), and TIM-3 (FITC, Biolegend, F38-2E2, 1:100).
IMC: CD3 (17Er, Fluidigm dilution 1:50), CD8A (162Dy, Fluidigm 1:100), Pan-cytokeratin (CK, Fluidigm 148Nd, 1:400), Granzyme B (167Er, Fluidigm 1:50), Ki67 (168Er, Fluidigm 1:100) and nucleic acid dye (191Ir/193Ir, Fluidigm 1:500)
IHC: CD3 (clone SP7, Thermo Fisher Scientific, MA, USA, 1:50), CD4 (clone 4B12, Thermo Fisher Scientific, MA, USA, 1:100,), CD8 (clone C8/144B, DAKO Glostrup Denmark, 1:200,)

Validation

All antibodies are commercially available and widely used in the scientific community.

The following antibodies were validated by the supplier:
- Actin (#sc-47778, Santa Cruz Biotechnology) has been validated by the supplier.
- pan-AKT (#2920, Cell Signaling) validated using siRNA, treatment with chemical activators and cell line comparison (https://www.cellsignal.de/about-us/our-approach-process/antibody-validation-western-blotting).
- pAKTS473 (#9271, Cell Signaling) validated using treatment with PDGF, wortmannin, LY294002. (https://www.cellsignal.de/about-us/our-approach-process/antibody-validation-western-blotting)
- cleaved Casp.3 (#9664, Cell Signaling) validated treatment with taurosporine and etoposide (https://www.cellsignal.de/products/primary-antibodies/cleaved-caspase-3-asp175-5a1e-rabbit-mab/9664)
- cPARP (#55-2597, BD Bioscience) validated using campotothecin treatment induce PARP cleavage (https://www.bdbiosciences.com/en-us/products/reagents/flow-cytometry-reagents/research-reagents/single-color-antibodies-ruo/purified-mouse-anti-cleaved-parp-asp214.552597)
- EGFR (#2239, Cell Signaling) validated using siRNA KO, treatment with chemical activators and cell line comparison https://www.cellsignal.de/about-us/our-approach-process/antibody-validation-western-blotting
- pEGFRY1068 (#3777, Cell Signaling, 1:1000) validated using EGF stimulation (https://www.cellsignal.de/products/primary-antibodies/phospho-egf-receptor-tyr1068-d7a5-xp-rabbit-mab/3777)
- ERK (#4696, Cell Signaling) validated using siRNA KO, treatment with chemical activators and cell line comparison (https://www.cellsignal.de/about-us/our-approach-process/antibody-validation-western-blotting)
- pERKT202/Y204 (#4370, Cell Signaling, 1:1000) validated using treatment with U0126 and TPA (https://www.cellsignal.de/products/primary-antibodies/phospho-p44-42-mapk-erk1-2-thr202-tyr204-d13-14-4e-xp-rabbit-mab/4370)
- H3K27me3 (#9733, Cell Signaling) validated using siRNA KO, treatment with chemical activators and cell line comparison (https://www.cellsignal.de/about-us/our-approach-process/antibody-validation-western-blotting)
- H3K9me3 (#C15410056, Diagenode) validated by the suppler using recombinant protein and whole cell extracts (https://www.diagenode.com/files/products/antibodies/Datasheet_H3K9me3_C15410056.pdf)
- total H3 (#C15310135, Diagenode) validated using whole cell extracts (https://www.diagenode.com/files/products/antibodies/Datasheet_H3pan_C15310135.pdf)
- Hsp90 (#4877, Cell Signaling) validated using siRNA KO, treatment with chemical activators or cell line comparison (https://www.cellsignal.de/about-us/our-approach-process/antibody-validation-western-blotting)
- IFIT1 (#14769, Cell Signaling) validated using treatment with human Interferon-α1 (https://www.cellsignal.de/products/primary-antibodies/ifit1-d2x9z-rabbit-mab/14769)
- IRF3 (#4302, Cell Signaling) validated using siRNA KO, treatment with chemical activators or cell line comparison (https://www.cellsignal.de/about-us/our-approach-process/antibody-validation-western-blotting)
- pIRF3S396 (#4947, Cell Signaling) validated using siRNA KO, treatment with chemical activators or cell line comparison (https://www.cellsignal.de/about-us/our-approach-process/antibody-validation-western-blotting)
- p16 (#ab108349, Abcam) validated using cell line comparison or knock-out cell lines (https://www.abcam.com/primary-antibodies/how-we-validate-our-antibodies#Western%20blot)
- p21 (#2947, Cell Signaling) validated using siRNA KO, treatment with chemical activators or cell line comparison (https://www.cellsignal.de/about-us/our-approach-process/antibody-validation-western-blotting)
- p65 (#8242, Cell Signaling) validated using siRNA KO, treatment with chemical activators or cell line comparison (https://www.cellsignal.de/about-us/our-approach-process/antibody-validation-western-blotting)
- pp65S5367 (#3033, Cell Signaling) validated using treatment with TNFa (https://www.cellsignal.de/products/primary-antibodies/phospho-nf-kb-p65-ser536-93h1-rabbit-mab/3033)
- RIG-I (#3743, Cell Signaling) validated using treatment with LPS (https://www.cellsignal.de/products/primary-antibodies/rig-i-d14g6-rabbit-mab/3743)
- TBK1 (#3504, Cell Signaling) validated using siRNA KO, treatment with chemical activators or cell line comparison (https://www.cellsignal.de/about-us/our-approach-process/antibody-validation-western-blotting)
- pTBK1S172(#5483, Cell Signaling) validated using treatment with LPS, phosphatase (https://www.cellsignal.de/products/primary-antibodies/phospho-tbk1-nak-ser172-d52c2-xp-rabbit-mab/5483)
- beta-2 microglobulin (B2M) (#A15770, ThermoFisher Scientific) validated clone using knock-down (https://www.thermofisher.com/antibody/product/MA1-19141.html)
- HLA-ABC (#11-9983-42, ThermoFisher Scientific) validated by relative expression (https://www.thermofisher.com/antibody/product/HLA-ABC-Antibody-clone-W6-32-Monoclonal/11-9983-42)
- CD3 (17A2, #46-0032-82; #100222) clone validated by relative expression (https://www.thermofisher.com/antibody/product/CD3-Antibody-clone-17A2-Monoclonal/14-0032-82)
- PD-1 (29F.1A12, #135210) validated using stimulation of splenocytes (https://www.biolegend.com/it-it/products/apc-anti-mouse-cd279-pd-1-antibody-6497)
- CD45 (30-F11, #103108) validated using relative expression (https://www.biolegend.com/en-us/products/fitc-anti-mouse-cd45-antibody-99?GroupID=BLG1932)

- CD3 (PE, Biolegend, UCHT1) validated using relative expression (https://www.biolegend.com/en-us/search-results/pe-anti-human-cd3-antibody-865)
- CD4 (APC, Biolegend, RPA-T4) clone validated using relative expression (https://www.biolegend.com/en-us/products/purified-anti-human-cd4-antibody-830?GroupID=BLG7755)
- CD69 (APC/Cy7, Biolegend,FN50) validated using cell stimulation (https://www.biolegend.com/en-us/search-results/apc-cyanine7-anti-human-cd69-antibody-1917)
- PD-1 (Pacific Blue, Biolegend, EH12.2H7) validated using cell stimulation (https://www.biolegend.com/en-us/search-results/pacific-blue-anti-human-cd279-pd-1-antibody-6153)
- PD-L1 (PE/Cy7, Biolegend, 29E.2.A3) validated using cell stimulation (https://www.biolegend.com/en-us/products/pe-cyanine7-anti-human-cd274-b7-h1-pd-l1-antibody-8277?GroupID=BLG5402)
- TIM-3 (FITC, Biolegend, F38-2E2) validated using cell stimulation (https://www.biolegend.com/fr-ch/products/fitc-anti-human-cd366-tim-3-antibody-9939)

The following antibodies were validated by us in addition to the supplier validation:
- BRAFV600E (#E19290, Spring Bioscience) was validated using BRAF V600E overexpression
- IRF1 (#8478, Cell Signaling) was validated using IRF1 overexpression
- MAVS (#3993, Cell Signaling) was validated using MAVS knock-out
- STING (#13647, Cell Signaling) was validated using STING knock-out
- NK1.1 (PK136, #25-5941-82) was validated using in vivo cell depletion
- CD8 (53-6.7, #100714) was validated using in vivo cell depletion
- p27 (#3686, Cell Signaling) was validated using p27 overexpression (data not shown)
- VTCN1 (#14572, Cell Signaling) and B7-H4/VTCN1 (#358104, BioLegend) were validated using VTCN1 overexpression (data not shown)
- Cas9 (#14697, Cell Signaling) was validated using Cas9 overexpression (data not shown)
- BCL2 (#2876, Cell Signaling) was validated using BCL2 overexpression (data not shown)
- MCL1 (#5453, Cell Signaling) validated using siRNA knock-down (data not shown)

IHC and IMC antibodies were validated by the Department of Pathology, University Hospital Cologne

Eukaryotic cell lines

Policy information about cell lines

Cell line source(s)	Human cancer cell lines PC9, HCC4006, HCC827, H3122, A375, H1993, SKBR3, BT474, Colo205 and HEK293T cells were purchased from ATCC. Murine PDAC cell lines were a gift from Prof. Siveke, 3LLC knock-out cells were a gift from Prof. Julian Downward.
Authentication	Cell lines were authenticated by STR profiling at the Institute of Forensic Medicine of the University Hospital of Cologne.
Mycoplasma contamination	All cell lines were routinely tested to be mycoplasma negative.
Commonly misidentified lines (See ICLAC register)	None of the cell lines were found among the frequently misidentified cell lines ICLAC v11.

Animals and other organisms

Policy information about studies involving animals; ARRIVE guidelines recommended for reporting animal research

Laboratory animals	Humanization experiments were performed in 8-10 week old male and female NSG mice (NOD.Cg-Prkdcscid Il2rgtm1Wjl/SzJ), for the syngeneic mouse model 6 week old female C57BL/6J mice were used. EGFR-mutant PDX models were propagated in female BALB/c nude mice aged 6-8 weeks at study start.
Wild animals	The study did not involve wild animals.
Field-collected samples	The study did not involve field-collected samples.
Ethics oversight	Animal experiments were approved by the institutional animal welfare committees of the University of Cologne (84-02.04.2017.A236) and the University of Okayama (OKU-2017412).

Note that full information on the approval of the study protocol must also be provided in the manuscript.

Human research participants

Policy information about studies involving human research participants

Population characteristics	PBMCs were obtained from healthy donors of the blood bank of the University of Cologne and population characteristics are not available. Lung adenocarcinoma PDXs had been established previously by Crown Biosciences Inc. and included one Asian female patient with an EGFR Exon 19 deletion and one Caucasian male patient with an EGFR L858R mutation.
Recruitment	PBMCs were obtained from healthy donors from the blood bank of the University of Cologne without additional clinical covariates for selection. Established PDX models were used based on known sensitivity to EGFR inhibitors.

Ethics oversight	Crown Bioscience, University of Cologne

Note that full information on the approval of the study protocol must also be provided in the manuscript.

Flow Cytometry

Plots

Confirm that:

- [x] The axis labels state the marker and fluorochrome used (e.g. CD4-FITC).
- [x] The axis scales are clearly visible. Include numbers along axes only for bottom left plot of group (a 'group' is an analysis of identical markers).
- [x] All plots are contour plots with outliers or pseudocolor plots.
- [x] A numerical value for number of cells or percentage (with statistics) is provided.

Methodology

Sample preparation	To assess lymphocyte composition in tumors and spleens of syngeneic EGFR-mut mice, spleens or tumor tiussue of mice were dissociated into single-cell suspensions Tumor Dissociation Kit, mouse (#130-096-730, Miltenyi Biotec) and a 70µm cell strainer (CORNING, USA). Then cells were stained with indicated fluorescence-labeled antibodies and subjected to Flow cytometric analysis. Cells were washed and incubated with mAbs for 30 min at 4 °C.<br><br>For Flow cytometry analyses of s.c. tumors in humanized mice, tissue of treated animals were harvested and mechanically dissociation using 40-mm cell strainers (BD Falcon). Red blood cells were lysed by ACK lysis buffer (Life Technologies), and cells were washed with PBS. Cells were stained for 30 minutes at 4°C prio to flow cytometry analysis.<br><br>For cell cycle analysis of cell lines, cells were harvested without supernatants by trypsinization, washed with cold PBS and fixed in 70% ethanol at -20°C. Subsequently cells were washed 2x with PBS, treated with RNAse and stained with Propidium Iodide for 1h at 37°C prior to flow cytometry analysis. To analyze dead cells by propidium iodide, supernatants of cells were harvested and adherent cells were brought into single cell suspension be detachment using trypsin. Both fractions per sample were joined, washed with PBS and stained with Priopidium Iodide (1µg/ml). For Annexin V/PI analysis cells and supernatants were harvested accordingly and washed with PBS. Samples were then resuspended in 100µL binding buffer with 0.5mg/ml Propidium Iodide and 2µL FITC-Annexin V. After incubation for 20min in the dark at room temperature staining was measured via flow cytometry analysis.
Instrument	FACS data were acquired on a BD Gallios 10/3 (Beckman Coulter, USA), LSR Fortessa (cat no. 647788, BS Bioscience) or a MACSquant Analyzer 16 (Miltenyi Biotec).
Software	Software used: Gallios software 1.2, Kaluza 2.1 (Beckman Coulter, Brea, CA, US), Diva software 8.0 (BD Bioscience), MACSQuantify v.2.13 and FlowJo software version 10.2 (TreeStar, Ashland, OR, USA).
Cell population abundance	Flow-cytometry based cell sorting was not performed, i.e. purity of post-sort samples was not investigated. Abundances of cell populations after gating is presented in the respective figures/source data files. Purity of the gated populations is high by definition and could not be further quantified using an independent method.
Gating strategy	In general, gating strategies contained a selection of single cells combined with further analyses of relevant markers. Details on the gating strategies for the different experimental set-ups can be found in Supplementary Fig. 10.<br><br>For cell cycle analyses debris and cell aggregates were removed based on TOF vs. PI Peak signals followed by gating on the PI signal to determine cell cycle phases. To determine dead cells by PI incorporation single cells were gated to exclude debris and cell aggregates based on FSC and SSC followed by gating on PI+ vs. PI- cells. Sequential gating to determine the fraction of exhausted T cells in tumors extracted from humanized mice was performed by selection of viable cells, inclusion of single cells based on FSC and SSC followed by gating on the respective cell population markers. Surface expression of proteins HLA, B2M, VTCN1 and PD-L1 was performed by exclusion of cell aggregates and debris using FSC and measurement of the respective fluorescent intensities. To determine lymphocyte subsets in spleens of syngeneic Egfrmut mice treated with osi+/-IVT4 it was gated on single cells followed by sequential gating on CD3+, then CD8+ or CD4+ cells followed by CD25/Foxp3. Gating strategy to determine amount of CD8 cells and PD-1 expression on CD8 T cells in tumors extracted from syngeneic Egfrmut mice by excluding dead cells, gating on single CD3 cells followed by determination of CD8+PD1+ cells. To determine amount of NK cells in tumors extracted from syngeneic Egfrmut mice dead cells and debris were excluded folled by determination of CD45+/NK1.1+/CD3- cells. To determine the amount of NK, CD4 and CD8 cells in spleens of syngeneic Egfrmut mice following depletion of cell populations single cells were determined and debris excluded followed by gating for CD3-/NK1.1+, CD3-/NK1.1+/CD49b+ or CD3+/CD8+ or CD3+/CD4+ cells.

- [x] Tick this box to confirm that a figure exemplifying the gating strategy is provided in the Supplementary Information.
